# “You could get the best of both breeds or the worst of both”: UK public attitudes towards crossbreeding in dogs - with a specific focus on brachycephalic dogs

**DOI:** 10.1371/journal.pone.0336661

**Published:** 2026-01-14

**Authors:** Elizabeth Youens, Dan G. O’Neill, Zoe Belshaw, Johanna Neufuss, Mickey S. Tivers, Rowena M. A. Packer

**Affiliations:** 1 Royal Veterinary College, Hertfordshire, United Kingdom; 2 EviVet Evidence-Based Veterinary Consultancy, Nottingham, United Kingdom; 3 Blue Cross, Burford, United Kingdom; 4 Paragon Veterinary Referrals, Wakefield, United Kingdom; University of Hawai'i at Manoa, UNITED STATES OF AMERICA

## Abstract

Extreme conformation and reduced genetic diversity are recognised to lead to severely reduced health, welfare and longevity in certain dog breeds. There is growing interest in applying strategic crossbreeding to promote more moderate conformations and greater genetic diversity within currently problematic breeds. Crossbreeding could therefore lead to more rapid and effective improvements in welfare compared to current practices of within-breed selection. Deliberate crossbreeding between distinct different dog breeds is not a new concept; it was historically commonly used to create the current pure breeds, to increase genetic diversity and to bring new physical and/or temperament traits into existing breeds. However, a recent surge in the popularity of ‘designer crossbreeds’ (intentional crosses between established purebreds) has elicited fresh interest around the potential positives and negatives of crossbreeding practices. Further research on crossbred brachycephalic dogs is urgently required for a greater understanding of the motivators and barriers to their acquisition. An online survey explored factors that motivate dog breed choice and acquisition of both crossbreed and purebred dogs. In addition, the survey used both closed and open questioning to explore the UK public’s perceptions of crossbreeding, specifically (i) between a brachycephalic breed and a non-brachycephalic breed, and (ii) between two non-brachycephalic breeds. Free-text results were analysed using content analysis and subsequently quantified. Results from 4,899 participants identified that key motivators to acquire a brachycephalic crossbreed vs a brachycephalic purebred included perceptions of improved health, including the reduction in risk of breed and conformation-related disorders, and increased genetic diversity. However, the desire to acquire a purebred dog, or even a specific breed, remained a significant barrier to crossbreed acquisition, alongside concerns surrounding the ethics of crossbreeding. Other barriers included perceived negative changes to appearance and temperament of the offspring from crossbreeding. The current study identified a common set of acquisition decision-making factors across all ownership groups, including desiring a dog who the owner perceives to enjoy being loved and to enjoy physical affection, but further demonstrated that good health is of motivational low priority to some dog owners, particularly to owners of purebred brachycephalic dogs. The mix of positive and negative public perceptions and beliefs around crossbreeding and crossbreed dogs demonstrate the need for further research into the health, temperament and appearance of brachycephalic crossbreed dogs. The suitability of crossbreed dogs as an alternative to certain current purebred breeds with high risk of genetic or conformational disorders depends on both public desire and on evidence-based selection of suitable breeds to encourage crosses which maximise canine welfare.

## Introduction

### The history of pedigree dog breeding

The genealogy of domestic dogs is culturally, socially and clinically important. Human perceptions about the relative merits of each dog’s heritage play major roles in dog acquisition decision-making [1–3] with the desire to own distinct breeds becoming increasingly globally popular over the last century [4,5]. Traditionally, the term ‘purebred’ describes animals from which both parents are considered to be of the same breed or variety [6]. Distinct breeds were invented and perpetuated through generations of controlled selective inbreeding (the mating of close relatives) within either relatively or absolutely closed populations [6]. Awareness of the genetic risks from close inbreeding – that was previously seen as good breeding practice – has now led some progressive Kennel Clubs to ban inbreeding for those closest relations, with the UK Royal Kennel Club introducing a ban in 2009 on registering the offspring from matings of first-degree relatives [7,8].

The concept of ‘pedigree’ dogs gained traction in the UK in the late 1800s. The Kennel Club was founded in 1873 and played a significant role in standardising dog breeds and recognising pedigree breeds [5]. ‘Pedigree’ dogs describes individuals from a pure breed that are registered with the relevant breed or kennel club and have several recorded generations of parentage history recorded [9]. Breed clubs started to form in the mid-19^th^ century with the goal of promoting the related concepts of breed differentiation and standardisation for their specific breed, and to begin the process of reproductive isolation of that breed as a sub-population within the wider domestic dog species [10]. Since then, many breeds have then gained recognition by one or more kennel clubs and registries globally and are given unique breed name terms that are internationally applied and recognised, alongside breed standards describing their desired physical and temperament characteristics [4].

### Health implications of pedigree dog breeding

Despite the popularity of purebred dogs, selective inbreeding for many purebreds has led to high levels of hereditary disease and breed conformations that negatively affect health [11], and therefore canine welfare, by impacting on an animal’s emotional state (e.g., by causing pain, discomfort, restrictions to natural expressions of behaviour and a reduced ability to have positive experiences) [12]. Modern pedigree dog breeding practices have faced growing criticism over recent decades for compromising dog health and welfare [13–17]. Many dog breeds now show significantly reduced genetic diversity as a result of genetic bottlenecks imposed by closed stud books [9]. For some breeds, there is mounting evidence on the increased odds of disorders directly related to these extreme breed-defining phenotypic features, such as muzzle length and skin folding [15].

A common example of extreme conformation in dogs is brachycephaly, a polygenetically inherited trait that has been selected for and exaggerated artificially by humans through years of selective inbreeding [18]. Brachycephaly in dogs is characterised by a mediolateral widening of the skull to produce a large, rounded head, paired with a rostrocaudal shortening of the muzzle, which can be severe [18]. There is now a large evidence base linking brachycephaly to increased risks of multiple severe and chronic disorders, including respiratory [19], ocular [20–23], dermatological [24], reproductive [25], spinal [26,27], and heat-related illness [28]. The profound disease burden associated with brachycephaly in dogs has necessitated entire textbooks dedicated to diagnosing, treating and palliating against disorders affecting most major body systems in dogs exhibiting this extreme conformation [29]. Several commonly owned brachycephalic breeds, such as the Pug, French Bulldog and English Bulldog, show increased odds of multiple conformational-related diseases [30–32] and are at risk of early death [33]. There is growing evidence that many current pure breeds of dogs carry substantial predispositions to disorders resulting from genetic or conformational problems, with almost 400 inherited disorders identified in dogs to date [15,34]. Therefore, there is an urgent need to reverse the harms caused by historical purebred breeding practices that led to extreme conformation and reduced genetic diversity in dogs.

### The history of crossbreeding

The UK Royal Kennel Club effectively closed their breed registers to crossbreeding in 1971. As a result, formally acknowledged and planned crossbreeding between different types of breeds of dogs to increase genetic diversity, reduce the risk of genetic disease and to moderate conformational exaggeration within breeds [35] was prevented as a breeding practice for pedigree dogs in the UK. However, in recent years, the UK Royal Kennel Club has relaxed this position in certain circumstances, and there is growing acceptance within breeding communities of formal crossbreeding programs designed for a specific purpose. For example, a Dalmatian-Pointer crossbreeding project was initiated to reduce problems associated with elevated levels of uric acid in the Dalmatian that could not be eliminated via within-breed selection [36,37]. In addition, a breeder-led Griffon Bruxellois-Australian Terrier crossbreeding project was initiated in the UK to reduce the prevalence of the Chiari malformation in the Griffon Bruxellois [38]. However, these examples are still relatively limited in comparison to the scale of inherited breed-related health disorders in the pedigree population [15], and some breed clubs refuse to acknowledge the legitimacy of dogs born outside of closed registers. Internationally, the pace of acceptance of crossbreeding projects is much more rapid in some countries: for example, systematic crossbreeding has been approved by the Finnish Kennel Club since 1997, with their first crossbreeding project between the Pinscher and Schnauzer, who were originally one breed [39]. The results were considered positive with increased health and desired characteristics of the Pinscher in the resulting cross [40].

### Crossbreeding for conformational health

The existence of hybrid vigour effects in domestic dogs as a result of crossbreeding has been debated [41], with the potential also being proposed for negative effects on the offspring compared to the progenitor parent breeds [42,43]. However, there is logical appeal to consider that the overall disease burden would reduce in offspring of dogs with extreme conformation compared to the extreme parent-breed if crossed with more moderate breeds, where most health issues are related to conformation: for example, in brachycephalic breeds crossed with non-brachycephalic breeds. To date, evidence on the health status of brachycephalic crosses is limited. It has been demonstrated that ‘Retropugs’ – a crossbreed of Pugs originally created in Germany via crossing purebred Pugs with Parson Russell Terriers – perform better than purebred Pugs in respiratory tests [44]. However, the small size of this study (n = 7 Retropugs and n = 42 Pugs) limits interpretation, but it seems likely there is potential for strategic crossbreeding to encourage more moderate conformations of the current extreme breed. This logic has been adopted internationally, and in 2023, separate crossbreeding projects for two brachycephalic breeds were accepted by the Finnish Kennel Club – the French Bulldog and Cavalier King Charles Spaniel [45] – in attempts to reduce brachycephalic obstructive airway syndrome (BOAS) and spinal issues in the former, and mitral valve disease and syringomyelia in the latter.

### Public acceptance of crossbreeding

The wider success of crossbreeding to potentially improve canine welfare by encouraging more moderate conformations requires widespread public acceptance of the concept and a high demand for these dogs, either as new crossbreeds (‘designers’) or as updated versions of current breeds (outcrossing). The social endorsement of owning a dog with purebred status and high predictability in future size, appearance and temperament in dogs are both seen as desirable traits for potential dog owners when deciding on a type of puppy to acquire [1–3]. This allure might explain an ongoing high demand for purebred dogs in the UK, where 69.4% of dogs are of a recognised breed [4]. However, times may be changing, with a recent study on the perceptions of Australian participants on their ideal dog revealing that participants mostly showed no preference regarding whether their dog was purebred or not (73%), with 19.5% preferring purebred dogs and 7.4% preferring mixed breed or designer (purposefully cross-bred) dogs [46].

However, using crossbreeding to create new types (i.e., broad categories of dogs based on form, function or style of work, lineage or appearance) and specific breeds of dog (i.e., closed groups of dogs sharing a common set of heritable characteristics) is not a new dog-breeding practice, with many of today’s purebreds originally ‘invented’ through various forms of intentional crossbreeding [43]. And now, a century later, the UK is currently witnessing a boom in ownership of ‘designer dogs’ [4] as a raft of newly invented breeds that are first-generation or later-generation intentional crosses between two (or more) purebred progenitor breeds [47]. The popularity of intentional crosses has risen sharply over the last few decades, especially those designer breeds with a Poodle progenitor such as the Labradoodle, and more recently the Cockapoo and Cavapoo [48,49].

In contrast to intentionally bred designer crossbreeds, dogs colloquially called ‘crossbreed’, ‘mongrel’ or ‘mutt’ are generally considered as non-intentional mixes of breeds or crossbreeds that may or may not be guessed at or known. In recent years, such dogs have been classified as ‘non-designer crossbreeds’ to differentiate them from other demographic groups [4].

### Acceptability of brachycephalic crossbreeding

In Sweden, a proposed introduction of planned crossbreeding to reduce the health issues of brachycephalic dogs garnered a very mixed response across veterinarians, dog breeders, dog owners, kennel clubs and breed groups [50]. Although all groups acknowledged the serious health problems associated with brachycephaly there was significant disagreement on which strategies should be followed to tackle these. Dog owners, breeders and vets were mostly in favour measures such as banning breeding of individual dogs with a short nose predisposed to clinical signs. In sharp contrast, show judges deviated from the other groups by preferring to follow existing pedigree breed standards, paradoxically even when it was the physical appearance promoted by the breed standard that was causing the health issues [50].

Understanding public perceptions of crossbreed dogs – and especially of brachycephalic crossbreed dogs – compared to purebred dogs is of high importance. Veterinary professionals, pet welfare charities, policy makers and other stakeholders in animal welfare need to be able to provide accurate information and advice on dog acquisition and breeding including recommending or deterring the public from certain breeds or conformations in an evidence-based manner. Preliminary evidence suggests current owners of designer crossbreeds are attracted to these newly ‘invented’ breeds based on perceptions of better health compared to their purebred progenitor breeds [47]. However, these data are largely derived from owners of Poodle-cross designer dogs and therefore further research is needed to understand whether these same drivers and perceptions apply to brachycephalic-crossbreed acquisition. Physical appearance has been demonstrated as another important factor driving acquisition of designer-crossbreed dogs, as well as being the leading factor in the acquisition of brachycephalic purebreds [51]. Given crossbreeding can result in changes to appearance, whether the offspring of brachycephalic crossbreeds are considered acceptable and desirable to prospective owners attracted to current extreme brachycephalic dogs is of high importance.

Furthermore, temperament – defined as the biological, instinctive part of personality which displays as an innate tendency to display certain traits [52] – is also a key decision-making factor explaining why owners choose to acquire and re-acquire specific brachycephalic breeds. This includes perceptions of these dogs as being good companions and possessing a ‘lazy’ temperament which requires little exercise, albeit the latter likely reflects exercise intolerance due to BOAS rather than a true temperament trait [51,53]. Given that the offspring of crossbreeds has been demonstrated to differ from their purebred progenitors, sometimes negatively from the owners’ perspective, in initial studies of Poodle-crosses [54], understanding expectations of temperament that may negatively affect likelihood of acquisition of crossbreed dogs is also vital, alongside quantification of potential behavioural differences between purebred and crossbreed brachycephalic dogs.

At present, there is little evidence on public perceptions of predictability, health, temperament and behaviour of crossbreed dogs, with no studies to date explicitly exploring these perspectives for brachycephalic crosses.

This study aimed to explore the UK public’s perceptions of the positives and negatives of crossbreeding and crossbreed dogs: both regarding brachycephalic breeds but also more generally. Furthermore, this study aimed to better understand acquisition-motivating factors for ownership of a variety of dog types, including differences between owners of brachycephalic and non-brachycephalic breeds, and between owners of purebred and crossbreed dogs.

## Materials and methods

An online questionnaire was developed to explore perceptions of crossbred dogs within the UK public. Ethical approval was granted from the Social Science Research Ethical Review Board at Royal Veterinary College (URN SR2023–0162). The questionnaire was designed iteratively between the authors and hosted using SurveyMonkey software. Participant inclusion criteria included being aged ≥18 years of age and a UK resident and was open to persons across the spectrum of dog ownership experience, from having no prior or planned dog ownership to also including past, present and prospective dog owners of all breeds and crossbreeds.

Participants were recruited using multiple pathways between 5^th^ January 2024–15^th^ February 2024. Snowball sampling used advertising posters (available in Supporting Information file 1) placed on various UK-based dog-focused groups on social media sites (Facebook and Instagram). Some UK veterinary practices were approached to engage their clients with the survey via social media and physical posters in waiting rooms. Pet industry stakeholders, including animal welfare charities and large veterinary organisations promoted the survey on their social media channels or by direct email. The survey link was emailed to n = 962 owners of Pugs and Pug-cross dogs who had already expressed interest or were actively engaged in a linked practical research study exploring the comparative health of brachycephalic purebred and crossbreed dogs and who had previously consented to being contacted about further research. The complete list of disseminators is shown in the Acknowledgements.

Participants gave written and informed consent that they:

- Were 18 years old or over.
- Were currently a resident of the UK.
- Had read and understood the above information and gave consent for their answers to be used for this research study and in any resulting publications.
- Gave permission for the anonymised data that cannot be traced to any individual to be deposited in the RVC research data storage facility and made available in association with subsequent publications so it can be used for future research and learning.

**Fig 1:**
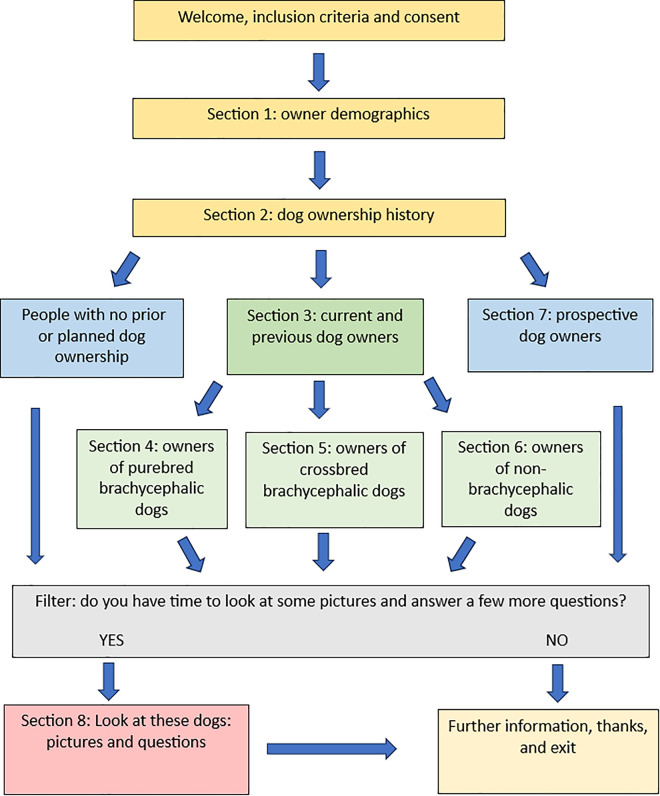
Survey Schematic. A schematic showing the structure of the survey exploring the UK public’s perceptions around crossbreeding and dog acquisition. All participants answered the first two sections and were then directed to relevant sections depending on their answers.

The survey comprised eight sections, with survey logic creating a bespoke experience for each participant based on their dog ownership history (Fig 1). Question types included closed-choice questions including binary yes/no, multiple choice and Likert scale questions, and open questions with free-text boxes to facilitate qualitative analyses. Participants could not amend previous answers once they had progressed to a new section.

The survey addressed:

- Acquisition-motivating factors for dog ownership, for quantitative comparison between three groups of dog owners (purebred brachycephalic dogs, crossbreed brachycephalic dogs and non-brachycephalic dogs)
- Free text responses surrounding positive and negative perceptions of cross-breeding, for qualitative content analysis
- Factors influencing future acquisition of brachycephalic or brachycephalic crossbreed dogs

Section 1 of the survey captured participant demographics, including age, gender and household membership, and section 2 captured canine demographics, including number and breed(s) of dog(s) currently and previously owned.

Participants were given a definition of brachycephaly and provided with a list of common brachycephalic dog breeds. The terms ‘purebred’ was defined as “both parents are of this same breed” and crossbreed was defined as “a mix between two or more purebreds”.

If the participants indicated in section 3 of the survey that they currently owned, or had previously owned, a purebred brachycephalic dog, they were subsequently filtered to section 4. Those participants who currently or previously owned a crossbreed brachycephalic dog were taken to section 5. Participants who owned more than one purebred or crossbreed brachycephalic dog were asked to use their most recently acquired dog as the basis of their responses. All other dog owners (i.e., owners of non-brachycephalic dogs, both purebred and crossbreed) were taken to section 6 and also asked to use their most recently acquired dog as the basis for their responses. Participants who didn’t currently, or hadn’t ever previously owned a dog, but were considering future acquisition were taken to section 7, with non-dog owning participants who had no future intention to acquire a dog were taken to section 8.

Sections 4–6 of the survey explored pre-acquisition motivations and potential future acquisition breed choices for each dog ownership group (owners of purebred brachycephalic dogs, owners of crossbreed brachycephalic dogs and owners of non-brachycephalic dogs). Participants were asked why they chose their dog that they were answering the question about, with a 5-point Likert scale (strongly disagree to strongly agree) used to rate each statement, e.g., ‘I wanted a breed I’d owned before’ and ‘I wanted a breed which needed lots of exercise’. Participants were also asked if they would consider owning the same breed again.

Attitudes towards intentional crossbreeding were assessed using free-text responses to four questions:

1. What, if anything, would you consider to be the *positives* of crossbreeding a pure-bred short-muzzled breed with any other pure-bred, but not short-muzzled, breed? E.g. a Pug crossed with a Jack Russell Terrier. Please explain in your own words in the box below.
2. What, if anything, would you consider to be the *negatives* of crossbreeding a pure-bred short-muzzled breed with any other pure-bred, but not short-muzzled, breed? Please explain in your own words in the box below.
3. What, if anything, would you consider to be the *positives* of crossbreeding ANY pure-bred dog (i.e., not limited to short-muzzled breeds) with a pure-bred dog from another breed? E.g. a Cocker Spaniel with a Poodle. Please explain in your own words in the box below.
4. What, if anything, would you consider to be the *negatives* of crossbreeding ANY pure-bred dog (i.e., not limited to short-muzzled) with a pure-bred dog from another breed? Please explain in your own words in the box below.

Section 8 involved participants scoring images of three brachycephalic breeds with varying degrees of extreme conformation on a number of domains; results from that section are not reported here and are included in a separate publication [55].

The full survey is available in Supporting Information file 2.

The survey was publicly open from 18^th^ January 2024–1^st^ March 2024. Participants could exit the survey at any time; however, because the survey was anonymous, respondents could not withdraw their response once submitted.

### Statistical methods

Survey data were exported from SurveyMonkey into a Microsoft Excel (2023) spreadsheet for cleaning. Responses from ineligible participants were excluded (those who did not progress past the initial introductory information and/or consent and duplicate responses). It was not possible to estimate how many persons were exposed to the survey link due to the nature of snowball sampling across multiple platforms, and therefore a formal response rate for the survey could not be estimated.

Descriptive and inferential data analysis was performed in IBM SPSS Statistics (V29.0.0.0). Descriptive statistics (frequency and percentage) were reported. One-way ANOVA was used for continuous data (including Likert scales) [56,57] to analyse variation in means between groups of dog ownership type. Pairwise comparison used Fisher’s least significant difference (LSD) post-hoc comparison when significant differences were detected between at least two groups.

A coding framework using content analysis was developed for each free-text question [58] and the data were then enumerated and analysed using the chi-square test to compare proportions between ownership groups. Representative written quotes were selected from groups of existing categories to provide further insights and illustrate the generated codes.

Statistical significance was set at the 5% level.

A range of experts and organisations were consulted from the authors’ professional networks during the design of the study and the recruitment of participants, including members of the funding organisations; however, the research team had total control over the final decisions on the study design, analysis and paper writing (decision to publish and content of the manuscript).

## Results

From 5,035 responses received, n = 115 (2.3%) responses were removed that held no data beyond consent and n = 21 (0.4%) responses were removed due to duplicate IP addresses, leaving n = 4,899 (97.3%) unique participants in the final analysis. Participants typically took 20–25 minutes to complete the survey.

### Participant demographics

The majority of participants self-described as female (n = 4,474, 91.3%), with n = 372 (7.6%) male, n = 24 (0.5%) agender or non-binary and n = 29 (0.6%) choosing ‘prefer not to say’. The median participant age group was 45–54 years with n = 1,095 (22.4%) of participants. Around one quarter of participants lived in households that included children, or had children visiting regularly (n = 1,179, 24.0%) and around one in six participants lived alone (n = 815, 16.6%). Over half (n = 2,865, 58.5%) of participants lived with other adults but no children. The most common type of home was a 3 + bedroom house (n = 3415, 69.7%). Participants who worked in the animal sector made up 20.0% (n = 978), within which n = 354 (36.0%) worked as companion animal veterinary surgeons or veterinary nurses.

### Dog ownership experience

The analysis included n = 4,730 (96.6%) participants who currently or previously owned a dog of any type and n = 169 (3.4%) participants who at the time of participating didn’t currently own, and had never owned, a dog of any type. Of those 4,730 participants who did or had owned a dog of any type, n = 1,270 (25.9%) participants currently or previously owned a purebred brachycephalic breed, and n = 429 (8.8%) currently or previously owned a crossbreed brachycephalic breed, while n = 3,031 (61.9%) currently or previously owned a non-brachycephalic breed. The most common number of dogs currently owned by dog-owning participants was one (n = 2,524, 51.5%), with 24.6% (n = 1,206) owning two. Of the participants who didn’t own a dog, and had never owned a dog at the time of participating in the questionnaire, n = 130 (2.7%) were considering owning one in the future, and n = 39 (0.8%) had no intention of owning one in future.

### Acquisition-motivating factors

Participants who currently or previously owned any dog type (n = 4730) were asked to rank common factors that motivated their choice of breed for that dog in acquisition.

The three most highly ranked acquisition-motivating factors in breed choice for owners of purebred brachycephalic dogs were: choosing a dog who enjoys being loved (mean score from 1 (strongly disagree) to 5 (strongly agree): 4.49, standard deviation (SD) 0.81); a dog who enjoys strokes/cuddles (mean 4.43, SD 0.82); and a dog whose size is suitable for their home/garden (mean 4.14, SD 1.06). The three most highly ranked acquisition-motivating factors in breed choice for owners of crossbreed brachycephalic dogs were: choosing a dog who enjoys being loved (mean 4.46, SD 0.84); a dog who enjoys strokes/cuddles (mean 4.41, SD 0.82); and a dog who is healthy (mean 4.28, SD 0.93). The three most highly ranked acquisition-motivating factors in breed choice for owners of non-brachycephalic dogs were: choosing a dog who was healthy (mean 4.49, SD 0.83); a dog who enjoys being loved (mean 4.41, SD 0.77); and a dog who enjoys strokes/cuddles (mean 4.29, SD 0.81).

The owners of non-brachycephalic dogs and crossbreed brachycephalic dogs scored several factors related to health and lifespan as stronger motivational factors for acquisition of their breed than owners of purebred brachycephalic dogs (Table 1). Owners of crossbreed brachycephalic dogs scored factors related to lower financial costs as stronger motivational factors for acquisition than owners of purebred brachycephalic dogs and non-brachycephalic dogs. A stronger motivational factor for current and past owners of purebred brachycephalic dogs than owners of crossbreed brachycephalic dogs and owners of non-brachycephalic dogs was choosing a dog which would be popular.

**Table 1:** One-way ANOVA* analysis of acquisition-motivating factors in breed choice for groups of owners of purebred brachycephalic, crossbreed brachycephalic and non-brachycephalic dogs. Highlighted in bold are results statistically significant between groups.

Breed choice motivator		Mean score from 1 (strongly disagree) to 5 (strongly agree) (Standard deviation)			F -statistic	p-value	Post-hoc comparison (LSD)** p-value		
Domain	Sub-category	Owners of purebred brachycephalic dogs (PB) (n = 675)	Owners of crossbreed brachycephalic dogs (XB) (n = 257)	Owners of non-brachycephalic dogs (NB) (n = 1124)			Purebred brachycephalic vs crossbreed brachycephalic	Purebred vs non-brachycephalic	Crossbreed brachycephalic vs non- brachycephalic
Previous ownership/ familiarity	I wanted a breed I’d owned before	2.93 (1.48)	2.45 (1.28)	3.25 (1.43)	58.79	<0.001	**<0.001**	**<0.001**	**<0.001**
	I wanted a dog like one I’d had while growing up	2.24 (1.29)	2.10 (1.17)	2.51 (1.28)	27.97	<0.001	0.089	**<0.001**	**<0.001**
	I wanted a dog like one that friends or family had	2.14 (1.27)	2.16 (1.23)	2.29 (1.26)	6.74	<0.001	0.787	**<0.001**	0.055
Cost of ownership	I wanted a dog with a low purchase cost	2.00 (1.08)	2.56 (1.57)	2.24 (1.11)	35.86	<0.001	**<0.001**	**<0.001**	**<0.001**
	I wanted a dog that had low upkeep costs	2.23 (1.10)	2.59 (1.19)	2.45 (1.12)	20.08	<0.001	**<0.001**	**<0.001**	**0.024**
Appearance	I wanted a dog I liked the look of	3.61 (1.27)	3.52 (1.27)	3.88 (1.10)	32.54	<0.001	0.231	**<0.001**	**<0.001**
Social status	I wanted a dog that other people liked the look of	2.07 (1.14)	2.25 (1.14)	2.32 (1.17)	17.54	<0.001	**0.009**	**<0.001**	0.313
	I wanted a popular breed	2.06 (1.03)	1.97 (1.08)	1.72 (0.98)	47.33	<0.001	0.119	**<0.001**	**<0.001**
	I wanted a dog who would reflect who I am to others	2.79 (1.25)	2.68 (1.14)	2.78 (1.21)	1.18	0.309	n/a	n/a	n/a
Exercise needs	I wanted a dog that didn’t need much exercise	2.52 (1.22)	2.41 (1.12)	1.96 (1.03)	111.33	<0.001	0.091	**<0.001**	**<0.001**
	I wanted a dog who needed lots of exercise	2.33 (1.08)	2.49 (1.13)	3.10 (1.18)	186.72	<0.001	**0.027**	**<0.001**	**<0.001**
Size	I wanted a dog whose size was suitable for my home/garden	4.14 (1.06)	4.26 (1.03)	4.18 (0.95)	1.89	0.151	n/a	n/a	n/a
Health/ longevity	I wanted a dog which would be healthy	3.95 (1.05)	4.28 (0.93)	4.49 (0.83)	136.84	<0.001	**<0.001**	**<0.001**	**<0.001**
	I wanted a dog with a longer life expectancy	3.42 (1.05)	3.66 (1.07)	3.80 (0.99)	55.28	<0.001	**<0.001**	**<0.001**	**0.014**
	I wanted a dog with a shorter life expectancy	1.63 (0.91)	1.57 (0.90)	1.46 (0.81)	15.54	<0.001	0.241	**<0.001**	**0.025**
Care needs	I wanted a dog which would be easy to look after	3.46 (1.00)	3.58 (0.97)	3.55 (0.92)	2.83	0.06	n/a	n/a	n/a
	I wanted a dog with high care needs	2.03 (0.91)	1.81 (0.86)	1.93 (0.91)	9.13	<0.001	**<0.001**	**0.002**	**0.021**
Behaviour	I wanted a dog which is calm	3.52 (0.98)	3.51 (0.91)	3.42 (0.96)	4.67	0.009	0.875	**0.050**	0.096
	I wanted a dog which is excitable	2.60 (0.98)	2.63 (0.94)	2.57 (0.98)	0.85	0.426	n/a	n/a	n/a
	I wanted a dog who would be safe with children	3.89 (1.17)	3.86 (1.10)	3.89 (1.09)	0.09	0.911	n/a	n/a	n/a
	I wanted a dog which would be easy to train	3.28 (0.95)	3.51 (0.92)	3.65 (0.94)	57.72	<0.001	**<0.001**	**<0.001**	**0.010**
Companionship	I wanted a dog which would make me laugh	3.73 (1.07)	3.64 (1.01)	3.70 (1.00)	0.86	0.423	n/a	n/a	n/a
	I wanted a dog who enjoys being loved	4.49 (0.81)	4.46 (0.84)	4.41 (0.77)	3.48	0.031	0.52	**0.01**	0.33
	I wanted a dog who enjoys strokes and/or cuddles	4.43 (0.82)	4.41 (0.82)	4.29 (0.81)	12.67	<0.001	0.68	**<0.001**	**0.009**

* Analysis of variance ** Least significant difference.

See Table 1 for full results on acquisition-motivating factors.

### Content analysis: attitudes towards cross-breeding

1. Positives of crossbreeding a purebred brachycephalic breed with a purebred non-brachycephalic breed

Improved health was cited as the most common positive for crossbreeding a brachycephalic breed with a non-brachycephalic breed for all ownership groups. This included references to non-specific health benefits, but also more specific health improvements such as an increased muzzle length and/or less-extreme facial conformation. A higher proportion of owners of crossbreed brachycephalic dogs cited less extreme facial conformation (*X*^2 ^= 11.77, p = 0.019) as positives of crossbreeding compared to other ownership groups. Specific mentions were made of certain disorders commonly associated brachycephaly in dogs, namely breathing, heat tolerance, skin conditions, eye protrusion, teeth, spinal and orthopaedic disorders (Table 2).

**Table 2:** Coded free text responses for survey participant answers on the positives of crossbreeding a purebred brachycephalic breed with any other purebred, but not brachycephalic, breed. Highlighted in bold are results statistically significant between groups.

Code		Ownership Type				*X* ^2^	p-value	Illustrative Quotes
		Purebred brachycephalic (PB) (n = 689)	Crossbreed brachycephalic (XB) (n = 280)	Purebred non-brachycephalic (PNB) (n = 1036)	Crossbreed non-brachycephalic (XNB) (n = 470)			
Improved health (non-specific)		229 (33.2%)	119 (42.5%)	385 (37.2%)	202 (43.0%)	14.28	**0.003**	“Reducing health issues” – Participant 4419 (PB) “Likely to have better health” – Participant 2832 (PNB)
Less extreme facial conformation		176 (25.5%)	100 (35.7%)	312 (30.1%)	130 (27.7%)	11.16	**0.011**	“Can help minimise the risks associated with the short-muzzled dog” – Participant 3784 (XB) “Hopefully the resultant cross would come out with a reasonably good length of muzzle” – Participant 2862 (PNB)
Improved health (specific problems related to brachycephaly):	Breathing	237 (34.4%)	96 (34.3%)	371 (35.8%)	177 (37.7%)	1.54	0.674	“I hoped that our dogs would not end up with the breathing problems associated with short muzzles” – Participant 486 (XB) “Issues such as poor breathing are reduced” – Participant 688 (XB)
	Heat tolerance	6 (0.9%)	7 (2.5%)	13 (1.3%)	3 (0.6%)	6.02	0.111	“The ability to better deal with heat” – Participant 131 (PB)
	Skin folds/ conditions	14 (2.0%)	4 (1.4%)	12 (1.2%)	8 (1.7%)	2.21	0.531	“Less skin wrinkling resulting in fewer yeast infections.” – Participant 835 (PB)
	Eye protrusion	19 (2.8%)	5 (1.8%)	15 (1.4%)	4 (0.9%)	6.88	0.076	“Less bulgy eye problems” – Participant 3870 (XB)
	Teeth	4 (0.6%)	5 (1.8%)	8 (0.8%)	3 (0.6%)	3.97	0.265	“Less dental overcrowding, less dental abnormalities.” – Participant 699 (PNB)
	Spinal	10 (1.4%)	6 (2.1%)	7 (0.7%)	1 (0.2%)	13.76	**0.003**	“Reduce spinal issues” – Participant 189 (PB)
	Orthopaedic	9 (1.3%)	5 (1.8%)	5 (0.5%)	1 (0.2%	12.98	**0.005**	“Remove genetic characteristics, e.g., bandy legs.” – Participant 411 (XNB)
Improved longevity		20 (2.9%)	16 (5.7%)	32 (3.1%)	15 (3.2%)	5.51	0.138	“I hear that hybrid dogs live longer” – Participant 54 (XNB) “Mixed breeding a short muzzle dog with a long muzzle dog will give the dog a longer life.” – Participant 1689 (XB)
Increased genetic diversity		39 (5.7%)	11 (3.9%)	42 (4.1%)	32 (6.8%)	6.48	0.09	“Lower COI [coefficient of inbreeding], increased genetic diversity” – Participant 4016 (PNB) “If the breed stock had a small gene pool or the existing stock was deviating from the breed standard, breeders might want to outcross to improve genetic diversity” – Participant 2726 (PNB)
Best of both breeds		5 (0.7%)	12 (4.3%)	25 (2.4%)	12 (2.6%)	13.22	**0.004**	“A cross breed has the best of both parents” – Participant 2063 (XNB) “To have favourable breed traits from both breeds” – Participant 492 (XB)
Good temperament		24 (3.5%)	47 (16.8%)	57 (5.5%)	26 (5.5%)	63.71	**<0.001**	“They have nice temperaments” – Participant 4335 (XNB) “The biggest positive for me was the temperament of my dog’s breed and if they were if a friendly nature, particularly with children” – Participant 755 (XB)
Pleasing appearance		6 (0.9%)	15 (5.4%)	29 (2.8%)	13 (2.8%)	17.07	**0.001**	“Cute looking” – Participant 397 (XNB) “Looks unique” – Participant 4822 (PB) “Nicer looking dogs normally” – Participant 1460 (PNB)
Ease of care:	Lack of shedding and perceived hypo-allergenicity	1 (0.1%)	10 (3.6%)	4 (0.4%)	9 (1.9%)	28.14	**<0.001**	“You can make dogs hypoallergenic who would otherwise not be without cross breeding.” – Participant 547 (XNB) I got the benefits of a Shih Tzu but because she is Poodle x Shih Tzu she is very low shedding and good for my allergies” – Participant 4039 (XB)
	Decreased purchase and insurance costs	2 (0.3%)	6 (2.1%)	1 (0.1%)	5 (1.1%)	19.43	**<0.001**	“Cheaper.” – Participant 1514 (XNB) “Decreased cost (upfront and ongoing)” – Participant 4811 (XNB)

Owners of crossbreed brachycephalic dogs were most likely to state that crossbreeding results in better temperament and improved appearance as positive reasons for crossbreeding compared to other ownership groups (temperament: *X*^2 ^= 64.46, p < 0.001, appearance: *X*^2 ^= 18.10, p = 0.001).

Perceived lack of shedding and hypo-allergenicity of crossbreed dogs was cited significantly more commonly by owners of crossbreed brachycephalic dogs (n = 10, 3.6%) and non-brachycephalic crossbreed dogs (n = 1, 1.9%) as a benefit of crossbreeding brachycephalic dogs, than owners of purebred brachycephalic dogs and purebred non-brachycephalic dogs (*X*^2 ^= 28.14, p < 0.001).

Perceived reduced costs (purchase and ongoing) as a benefit of crossbreeding brachycephalic dogs were most commonly cited by owners of crossbreed brachycephalic dogs (n = 6, 2.1%) and non-brachycephalic crossbreed dogs (n = 5, 1.1%) compared to owners of purebred brachycephalic dogs and purebred non-brachycephalic dogs (*X*^2 ^= 19.43, p < 0.001).

2. Negatives of crossbreeding a purebred brachycephalic breed with a purebred non-brachycephalic breed

A commonly cited negative effect of crossing a brachycephalic breed with a non-brachycephalic breed was the perception of detrimental effects on health (Table 3). General worsening of health was cited by participants from all ownership groups with no significant differences in frequency of expression. A particular concern regarding health was the risk of introducing the brachycephalic phenotype which might potentially worsen the health of the offspring in comparison to the non-brachycephalic progenitor. This concern was cited statistically less frequently (*X*^2 ^= 112.41, p < 0.001) by of owners of purebred brachycephalic dogs (n = 57, 9.6%), compared to all other ownership groups (owners of crossbreed brachycephalic dogs: n = 68, 29.4%, owners of purebred non-brachycephalic dogs: n = 305, 28.3%, owners of crossbreed non-brachycephalic dogs: n = 145, 34.4%).

**Table 3:** Coded free text responses for survey participant answers on the negatives of crossbreeding a pure-bred brachycephalic breed with any other pure-bred, but not brachycephalic, breed. Highlighted in bold are results statistically significant between groups.

Code		Ownership Type				*X* ^2^	p-value	Illustrative quotes
		Purebred brachycephalic (PB) (n = 591)	Crossbreed brachycephalic (XB) (n = 231)	Purebred non-brachycephalic (PNB) (n = 1079)	Crossbreed non-brachycephalic (XNB) (n = 421)			
Detrimental effects on health	Crossbreeding itself causes health issues	145 (23.5%)	47 (20.3%)	206 (19.8%)	78 (19.5%)	8.28	0.061	“Breeding new health issues between breeds” – Participant 820 (PNB) “Too much cross breeding can lead to health problems” – Participant 1709 (XNB)
	Adversely affect the health of the offspring by introducing the brachycephalic phenotype	57 (9.6%)	68 (29.4%)	305 (28.3%)	145 (34.4%)	112.41	**<0.001**	“The problems of having a short muzzle could be transferred to a usually non short muzzled breed” – Participant 889 (PNB) “Could be breeding brachy features into non-brachy breeds” – Participant 534 (XNB)
Detrimental impacts on temperament and/or personality		123 (20.8%)	21 (9.1%)	80 (7.4%)	22 (5.2%)	93.22	**<0.001**	“Temperament could change undesirably, e.g., less cuddly, more yappy, harder to train, more prey drive” – Participant 2788 (PNB) “Crossbreeding has a detrimental effect on the dog’s personality” – Participant 185 (PB)
Detrimental effects on appearance		35 (5.9%)	8 (3.5%)	26 (2.4%)	8 (1.9%)	17.96	**<0.001**	“You lose the aesthetics of the breed you want” – Participant 1308 (PB) “The dog no longer looks as attractive as it was if it was pure bred. I find pure bred pugs to be much cuter than cross breeds, e.g., Jugs” – Participant 4174 (PB)
Unpredictable effects on temperament and/or appearance		72 (12.2%)	21 (9.1%)	141 (13.1%)	40 (9.5%)	5.56	0.135	“Unpredictable traits” – Participant 653 (XNB) “No to cross breeding as not too sure what you will get as they grow into themselves” – Participant 2885 (PNB)
Loss of pedigree and/or purity of genes		88 (14.9%)	12 (5.2%)	59 (5.5%)	17 (4.0%)	61.46	**<0.001**	“Ruining the preservation of purebred dogs that have been around for years.” – Participant 3378 (PNB) “Mutts are a genetic disaster” – Participant 2857 (PB) “Diluting the pedigree standards” – Participant 667 (PNB)
Worst of both breeds		15 (2.5%)	5 (2.2%)	55 (4.1%)	16 (3.8%)	8.87	**0.031**	“Can mix the flaws in both breeds into one dog” – Participant 4161 (PB) “The pups may have inherited the negative attributes from both Damn and Sire” – Participant 2162 (PB)
Breeders’ motivations for crossbreeding are for fashion and/or financial gain only		46 (7.8%)	20 (8.7%)	99 (9.2%)	33 (7.8%)	4.19	0.651	“Sadly many people are crossing any breeds with any others, making up silly names and selling the puppies for ridiculous prices.” – Participant 1681 (PB) “I do not approve of dogs being bred to have cute names or to meet the demands of fashion” – Participant 115 (PNB)
No health testing or monitoring schemes		15 (2.5%)	2 (0.9%)	19 (1.8%)	3 (0.7%)	6.06	0.111	“No monitoring or standard” – Participant 946 (PB) “There’s no health testing schemes for crossbreeds” – Participant 2434 (XNB)
Possibility of crossing incompatible breeds		26 (4.6%)	8 (3.6%)	66 (6.1%)	21 (4.4%)	5.23	0.076	“Not breeding with a similar breed, causing issues” – Participant 2601 (XNB) “Some breeds are totally unsuitable to be crossed together” – Participant 809 (PB)
General negative attitude to breeding		11 (1.9%)	5 (2.2%)	26 (2.4%)	12 (2.8%)	1.12	0.772	“Too many dogs needing homes in this country, so would not add to the dog population” – Participant 104 (XNB) “There are enough dogs that need homes” – Participant 3383 (XNB)
General negative attitude to crossbreeding		20 (3.4%)	0 (0.0%)	37 (3.4%)	10 (2.4%)	8.94	**0.030**	“You shouldn’t cross breed animals, I think it can cause problems.” – Participant 1046 (PB) “I don’t like cross breeding, human interference is never good” – Participant 3148 (PB)

Owners of purebred brachycephalic dogs were most likely (*X*^2 ^= 93.22, p < 0.001) to cite potential detrimental effects of crossing brachycephalic with non-brachycephalic breeds on temperament and/or personality, with 1 in 5 (n = 123, 20.8%) owners expressing this concern compared to 1 in 10 or fewer owners of crossbreed brachycephalic dogs (n = 21, 9.1%), owners of purebred non-brachycephalic dogs (n = 80, 7.4%) and owners of crossbreed non-brachycephalic dogs (n = 22, 5.2%).

Although relatively rarely cited, owners of purebred brachycephalic dogs were also most likely (*X*^2 ^= 17.96, p < 0.001) to cite a detrimental effect on appearance (n = 35, 5.9%) as a result of crossing brachycephalic with non-brachycephalic dogs compared to owners of crossbreed brachycephalic dogs (n = 8, 3.5%), owners of purebred non-brachycephalic dogs (n = 26, 2.4%), and owners of crossbreed non-brachycephalic dogs (n = 8, 1.9%).

Owners of purebred brachycephalic dogs were also most likely (*X*^2 ^= 61.46, p < 0.001) to state the loss of pedigree or purity of the genes (n = 88, 14.9%) as a negative of crossing brachycephalic with non-brachycephalic dogs compared to owners of crossbreed brachycephalic dogs (n = 12, 5.2%), owners of purebred non-brachycephalic dogs (n = 59, 5.5%), and owners of crossbreed non-brachycephalic dogs (n = 17, 4.0%).

3. Positives of crossbreeding ANY purebred dog (i.e., not limited to brachycephalic breeds) with any other purebred dog from another breed

As observed for breeding brachycephalic with non-brachycephalic dogs, health was again cited as a positive reason for crossing any purebred breed with another purebred breed, alongside other quality and quantity of life factors such as greater longevity and the reduction of breed-specific disease (Table 4).

**Table 4:** Coded free text responses for survey participant answers on the positives of crossbreeding any purebred dog with any other purebred dog. Highlighted in bold are results statistically significant between groups.

Code		Ownership Type				*X* ^2^	p-value	Illustrative quotes
		Purebred brachycephalic (PB) (n = 584)	Crossbreed brachycephalic (XB) (n = 225)	Purebred non-brachycephalic (PNB) (n = 1236)	Crossbreed non-brachycephalic (XNB) (n = 535)			
Greater longevity		19 (3.3%)	9 (4.0%)	32 (2.6%)	28 (5.2%)	8.211	**0.042**	“Cross breeds generally live longer” – Participant 1629 (PNB)
Health	Improved general health	148 (25.3%)	72 (32.0%)	209 (16.9%)	143 (26.7%)	42.274	**<0.001**	“Better overall health” – Participant 829 (XNB) “I understand that cross breeds are generally healthier than pure breeds.” – Participant 807 (XNB)
	Improved health, but not a priority	9 (1.5%)	0 (0.0%)	14 (1.1%)	9 (1.7%)	4.213	0.239	“None, why would you other than for health reasons?” – Participant 959 (PNB) “I’m not sure I can think of any advantages. I suppose health.” Participant 1607 (PB)
	Reduction of breed-specific predispositions	129 (22.1%)	59 (26.2%)	280 (22.7%)	140 (26.2%)	4.171	0.244	“Lowering risk of health issues associated with specific pure breeds.” – Participant 2639 (XNB) “You can try and breed out the health problems that the breeds have” – Participant 1228 (PB)
Offspring conferred the best of both breeds		44 (7.5%)	29 (12.9%)	125 (10.1%)	75 (14.0%)	13.964	**0.003**	“To have favourable breed traits from both breeds” – Participant 491 (XB) “Getting the best breed traits of both breeds” – Participant 716 (XNB)
Good temperament		52 (8.9%)	27 (12.0%)	107 (8.7%)	77 (14.4%)	15.354	**0.002**	“I think the biggest advantage is the temperament of the dog. Most are so placid.” – Participant 402 (XNB) “The positives for me are the temperament of the cross breed dogs.” – Participant 755 (XB)
Improved genetic diversity		75 (12.8%)	26 (11.6%)	183 (14.8%)	88 (16.4%)	4.607	0.203	“Increasing gene pool in breeds with small numbers.” – Participant 994 (PNB)
Development of a new, unique, breed		43 (7.4%)	25 (11.1%)	39 (3.2%)	13 (2.4%)	43.044	**<0.001**	“May fill a niche that either breed couldn’t fulfil” – Participant 3384 (PB) “You could create a new breed with traits desired by yourself” – Participant 2026 (PNB)
Decreased purchase and insurance costs		8 (1.4%)	1 (0.4%)	3 (0.2%)	2 (0.4%)	9.788	**0.020**	“Having the advantage of not costing so much, e.g., insurance” – Participant 2762 (XNB) “Cheap pups” – Participant 4428 (PB)
Lack of shedding and perceived hypo-allergenicity		57 (9.8%)	27 (12.0%)	140 (11.3%)	95 (19.6%)	25.611	**<0.001**	“You can get dogs from this type of mating that are non-shedding so good for owners with allergies” – Participant 4294 (XNB)
Smaller size		5 (0.9%)	6 (2.7%)	11 (0.9%)	10 (1.9%)	7.441	0.069	“The opportunity to reduce the size of a breed you love.” - Participant 1868 (XNB)
Specific use, e.g., working, guide dogs, agility		9 (1.5%)	9 (4.0%)	34 (2.8%)	18 (3.4%)	5.347	0.148	“For specific reasons like guide dog crosses” – Participant 1095 (PNB) “Create a particular blend of characteristics for a particular sport or work (e.g., the Eurohound for canicross)” – Participant 1367 (XNB)
Pleasing appearance		11 (1.9%)	13 (6.1%)	34 (2.8%)	37 (6.9%)	22.461	**<0.001**	“Cute looking dogs” – Participant 1577 (XB) “You can get a fun looking dog” – Participant 682 (XNB)

General improvements to health as a result of generic purebred to purebred crossing was stated most commonly by owners of crossbreed brachycephalic dogs (n = 72, 32.0%) compared to owners of purebred brachycephalic dogs (n = 148, 25.3%), purebred non-brachycephalic dogs (n = 209, 16.9%), and crossbreed non-brachycephalic dogs (n = 143, 26.7%).

Owners of crossbreed non-brachycephalic dogs were significantly more likely to state that crossbreeding any purebred breed to any other purebred breed would result in offspring being conferred the ‘best of both breeds’ (*X*^2 ^= 13.964, p = 0.003), an improved temperament (*X*^2 ^= 15.354, p = 0.002), improved appearance (*X*^2 ^= 22.461, p < 0.001) and the lack of shedding/perceived hypoallergenic nature of crossbreeds (*X*^2 ^= 25.611, p < 0.001) compared to all other ownership groups.

4. Negatives of crossbreeding ANY purebred dog (i.e., not limited to brachycephalic breeds) with any other purebred dog from another breed

As observed with perceptions of crossing brachycephalic with non-brachycephalic dogs, health was again commonly cited as a negative of crossbreeding any purebred dog with any other purebred dog (Table 5). A detrimental effect of crossbreeding on general health was significantly more likely to be stated by owners of purebred brachycephalic dogs compared to all other ownership groups (*X*^2 ^= 18.668, p < 0.001). Within this broad category, a specific health concern of crossbreeding was the potential to exacerbate existing breed-specific disease, which was cited by all ownership groups equally (*X*^2 ^= 0.716, p = 0.869).

**Table 5:** Coded free text responses for survey participant answers on the negatives of crossbreeding any purebred dog with any other purebred dog. Highlighted in bold are results statistically significant between groups.

Code		Ownership Type				*X* ^2^	p-value	Illustrative quotes
		Purebred brachycephalic (PB) (n = 601)	Crossbreed brachycephalic (XB) (n = 206)	Purebred non-brachycephalic (PNB) (n = 1056)	Crossbreed non-brachycephalic (XNB) (n = 456)			
Health	Detrimental effect on general health	107 (17.8%)	29 (14.1%)	128 (12.1%)	42 (9.2%)	18.668	**<0.001**	“Possible health issues” – Participant 218 (PNB) “Detrimental to overall health” – Participant 591 (PB)
	Worst of both breeds/ exacerbate or compound existing breed problems	98 (16.3%)	30 (14.6%)	169 (16.0%)	78 (17.1%)	0.716	0.869	“Bring out the worst of both the breeds” – Participant 813 (PB) “Producing a dog with serious heritable health conditions from both types of pure-bred parents” – Participant 952 (XNB) “Potential negative qualities combined or amplified” – Participant 20 (PNB)
	Future harm from genetic changes	29 (4.8%)	13 (6.3%)	37 (3.5%)	24 (5.3%)	4.817	0.186	“Risk of potentially harmful genetic changes” – Participant 2832 (PB) “My worry is that the resulting puppies may have genetic problems” – Participant 1160 (PNB)
Detrimental effect on temperament		71 (11.8%)	19 (9.2%)	89 (8.4%)	39 (8.6%)	5.674	0.129	“Cross breeds can create worse temperaments” -Participant 1968 (PNB) “Could change temperament of the breed” – Participant 2457 (PB)
Breeders’ motivations for crossbreeding are for fashion and/or financial gain only		82 (13.6%)	40 (19.4%)	136 (12.9%)	48 (10.5%)	9.997	**0.019**	“Breeding dictated by ‘fashion’ and popularity trends” – Participant 2639 (PB) “Trying to cash in on the “designer dog” rush. No consideration of health, welfare and temperament” – Participant 104 (PNB)
Possibility of crossing incompatible breeds		47 (7.8%)	22 (10.7%)	70 (6.6%)	36 (7.9%)	4.317	0.229	“Think should be limited to similar types of dogs as so many breeds don’t work with each other” – Participant 97 (PB) “Many of the breeds are not compatible which causes problems” – Participant 4224 (PNB)
No health testing or monitoring schemes		21 (3.5%)	4 (1.9%)	37 (3.5%)	5 (1.1%)	8.088	**0.044**	“Less likely for health tests from parents” – Participant 3632 (PB) “There is usually a lack of health testing” – Participant 4333 (PNB)
Loss of breeds and/or breed identity		63 (10.5%)	4 (1.9%)	74 (7.0%)	21 (4.6%)	23.353	**<0.001**	“I worry about the decline and maybe loss of pure bred ‘unpopular’ dogs” – Participant 472 (PNB) “Losing the identity of individual breeds” – Participant 1928 (PNB)
Unpredictable effects on temperament and/or appearance		100 (16.6%)	41 (19.9%)	211 (20.0%)	73 (16.0%)	5.053	0.168	“Unexpected and varying results” – Participant 2056 (PNB) “Not sure what you’re getting” – Participant 4242 (XB)
High purchase cost		7 (1.2%)	5 (2.4%)	22 (2.1%)	6 (1.3%)	2.962	0.397	“These designers can be very expensive” – Participant 541 (XNB)
General negative attitude to breeding		18 (3.0%)	5 (2.4%)	23 (2.2%)	16 (3.5%)	2.525	0.471	“I am generally not in favour of breeding as long as there is already a massive surplus of dogs in shelters, rescue centre, dog pounds” – Participant 482 (XNB)

Owners of purebred brachycephalic dogs (n = 45, 7.5%) and owners of purebred non-brachycephalic dogs (n = 47, 4.5%) were significantly (*X*^2 ^= 31.07, p < 0.001) more likely to be against crossbreeding generally compared to owners of crossbreed brachycephalic and crossbreed non-brachycephalic dogs.

Irresponsible breeding practices were cited as a common negative perception of crossbreeding any purebred dog with any other purebred dog, with this category sub-divided into three main areas of concern: breeding for fashion and/or financial gain (total across ownership groups n = 306, 13.2%), the crossbreeding of incompatible breeds (total across ownership groups n = 175, 7.5%) and the lack of health tests available for crossbreed dogs (total across ownership groups n = 67, 2.9%).

Owners of purebred brachycephalic dogs were significantly more likely to cite the complete extinction of a breed or loss of a breed identity as a negative to crossbreeding any purebreds (n = 63, 10.5%, *X*^2 ^= 23.353, p < 0.001) compared to all other ownership groups (crossbreed brachycephalic dogs: n = 4, 1.9%; purebred non-brachycephalic dogs: n = 74, 7.0%; crossbreed non-brachycephalic dogs: n = 21, 4.6%).

### Future acquisition of dogs

Owners of non-brachycephalic breeds were significantly more likely to consider owning the same breed again than owners of purebred brachycephalic or cross-breed brachycephalic dogs (owners of purebred brachycephalic dogs: n = 550, 62.8% vs owners of crossbreed brachycephalic dogs: n = 159, 48.6% vs owners of non-brachycephalic dogs n = 1785, 70.4%; *X*^2^ = 120.89, df = 4, *p* < 0.001) (Table 6).

**Table 6:** Future dog acquisition plans of owners of purebred brachycephalic, crossbreed brachycephalic and non-brachycephalic dogs. Highlighted in bold are results which are statistically significant.

Future dog acquisition plans	Answer	Purebred brachycephalic owners (n = 876)	Crossbreed brachycephalic owners (n = 327)	Non-brachycephalic owners (n = 2537)	*X* ^2^	p-value
Would you consider the *same* breed/ crossbreed again?	Yes	550 (62.8%)	159 (48.6%)	1785 (70.4%)	120.891	**<0.001**
	No	137 (15.6%)	54 (16.5%)	161 (6.3%)		
	Don’t know/unsure	189 (21.6%)	114 (34.9%)	591 (23.3%)		
Would you consider a *purebred* brachycephalic dog?	Yes	429 (48.8%)	73 (22.3%)	146 (5.8%)	1040.079	**<0.001**
	No	291 (33.1%)	186 (56.7%)	2170 (85.5%)		
	Don’t know/unsure	160 (18.2%)	69 (21.0%)	222 (8.7%)		
Would you consider a *crossbreed* brachycephalic dog?	Yes	486 (55.2%)	170 (51.8%)	419 (16.6%)	622.099	**<0.001**
	No	231 (26.3%)	71 (21.6%)	1517 (59.9%)		
	Don’t know/unsure	163 (18.5%)	87 (26.5%)	595 (23.5%)		

Owners of purebred brachycephalic dogs were significantly more likely to consider acquiring a *purebred* brachycephalic dog in future, with half (n = 429, 48.8%) stating that they would, compared to around one in five (n = 73, 22.3%) owners of crossbreed brachycephalic dogs and around 1 in twenty (n = 146, 5.8%) owners of non-brachycephalic dogs (*X*^2^ = 1040.08, df = 4, *p* < 0.001).

Around half of owners of both purebred brachycephalic dogs (n = 486, 55.2%) and crossbreed brachycephalic dogs (n = 170, 51.8%) stated that they would consider owning a crossbreed brachycephalic dog in the future, which was significantly higher (*X*^2^ = 622.10, df = 4, *p* < 0.001) than for owners of non-brachycephalic dogs (n = 419, 16.6%).

1. Reasons for considering acquisition of a crossbreed brachycephalic dog

Reasons cited for considering acquiring a crossbreed brachycephalic dog included that the offspring were perceived to have improved health compared to the brachycephalic progenitor breed (Table 7).

**Table 7:** Coded free text responses for survey participant answers on why they would consider acquiring a crossbreed dog where one parent is a brachycephalic breed. Highlighted in bold are results statistically significant between groups.

Code	Ownership Type				*X* ^2^	p-value	Illustrative quotes
**Purebred brachycephalic (PB)** **(n = 317)**	**Crossbreed brachycephalic (XB) (n = 171)**	**Purebred non-brachycephalic (PNB) (n = 203)**	**Crossbreed non-brachycephalic (XNB) (n = 145)**
Improved health compared to the brachycephalic progenitor	115 (36.3%)	40 (23.4%)	73 (36.0%)	45 (31.0%)	9.745	**0.021**	“Knowing it could improve the dogs health and life expectancy” – Participant 3663 (PB) “I would consider a chihuahua cross in future if I felt the cross resulted in a healthy happy dog.” – Participant 336 (PB)
Previous positive experience with, or desire for brachycephalic dogs	40 (12.6%)	35 (20.5%)	12 (5.9%)	7 (4.8%)	26.944	**<0.001**	“I would consider owning a cross breed dog with one parent being a short muzzled breed as I have had a great experience with my pug and know many others who have.” – Participant 313 (PB) “I like the characteristics and looks of short muzzled dogs, have grown up with them so know their personalities well so if it’s nature and exercise demands was still “brachy like” then I’d be happy to consider a cross” Participant 2137 (PB)
Only acquire if rescued or rehomed, would not purchase	70 (22.1%)	30 (17.5%)	80 (39.4%)	60 (41.4%)	39.947	**<0.001**	“I would consider rescuing a cross-breed short-muzzled breed but not buying one” – Participant 3544 (XNB) “I wouldn’t actively seek out a short muzzled cross breed. I would only consider if it was a rescue “ - Participant 2394 (XNB)
Perceived benefits of crossbreeds more generally	23 (7.3%)	27 (15.8%)	5 (2.5%)	25 (17.2%)	31.315	**<0.001**	“I like cross breeds and consider them healthier overall” – Participant 4016 (XNB) “I think crossing them is a great idea” – Participant 4688 (XB)
Would consider most dogs – breed is less important than individual	99 (31.2%)	61 (35.7%)	65 (32.0%)	38 (26.2%)	3.300	0.348	“Breed isn’t the most important thing when choosing a dog” – Participant 4096 (XNB) “Providing I can give the care and love a dog needs, I would own/help majority of breeds. I’m a fan of many breeds.” – Participant 1587 (XB)

Owners of crossbreed brachycephalic dogs were significantly more likely to state that they would consider acquiring a brachycephalic cross due to previous positive experience of a brachycephalic breed (n = 35, 20.5%) compared to other ownership groups (*X*^2 ^= 26.944, p < 0.001).

Owners of both non-brachycephalic breed groupings (purebred non-brachycephalic: n = 80, 39.4% and crossbreed non-brachycephalic: n = 60, 41.4%), were statistically more likely (*X*^2 ^= 39.947, p < 0.001) to state they would only acquire a brachycephalic crossbreed if it came from a rescue or was rehomed, rather than purchased, compared to owners of purebred brachycephalic dogs (n = 70, 22.1%) and crossbreed brachycephalic dogs (n = 30, 17.5%).

2. Reasons against considering acquisition of a crossbreed brachycephalic dog

The desire to only own a purebred dog was significantly more commonly cited by owners of purebred dogs (purebred brachycephalic: n = 93, 35.4; purebred non-brachycephalic dogs: n = 108, 15.4%) compared to owners of crossbreed dogs (crossbreed brachycephalic: n = 2, 4.9%; crossbreed non-brachycephalic: n = 6, 2.6%) (*X*^2 ^= 103.454, p < 0.001).

Other cited reasons for not considering acquiring a brachycephalic crossbreed dog include potential health problems (overall cited n = 688, 55.6%), unpredictability (overall cited n = 73, 5.9%), disliking their temperament (overall cited n = 31, 2.5%) or appearance (overall cited n = 72, 5.8%) and the potential to encourage unethical breeding practices (overall cited n = 111, 9.0%) (Table 8).

**Table 8:** Coded free text responses for survey participant answers on why they would not consider acquiring a crossbreed dog where one parent is a brachycephalic breed. Highlighted in bold are results statistically significant between groups.

Code	Purebred brachycephalic (PB) (n = 263)	Crossbreed brachycephalic (XB) (n = 41)	Purebred non-brachycephalic (PNB) (n = 700)	Crossbreed non-brachycephalic (XNB) (n = 234)	*X* ^2^	p-value	Illustrative quotes
Potential health problems	98 (37.3%)	27 (65.9%)	386 (55.1%)	177 (75.6%)	75.692	**<0.001**	“Higher risk of health issues” – Participant 2059 (PNB) “They have the potential to have poor health from their brachycephalic parent” – Participant 2439 (XNB)
Strong preference for purebred dogs	93 (35.4%)	2 (4.9%)	108 (15.4%)	6 (2.6%)	103.454	**<0.001**	“I will NEVER own a cross breed dog when well bred purebred dogs are bred for health and conformation” – Participant 2459 (PB) “I own pure breed dogs so don’t want a mongrel” – Participant 985 (PNB)
Unpredictable effects on temperament and/or appearance	32 (12.2%)	6 (14.6%)	30 (4.3%)	5 (2.1%)	33.513	**<0.001**	“My main issue with cross breeding is unpredictability, you don’t know what the genetics will lead to when you breed two completely different breeds together.” – Participant 742 (PNB)
Encourages unethical breeding	20 (7.6%)	4 (9.8%)	57 (8.1%)	30 (12.8%)	5.469	0.140	“I don’t want to encourage breeding for greed and fashion trends.” – Participant 2075 (XNB)
Dislike their temperaments	13 (4.9%)	1 (2.4%)	13 (1.9%)	4 (1.7%)	8.214	**0.042**	“I don’t really like their temperaments” – Participant 2024 (PNB) “I’ve met a really snappy one” – Participant 757 (XNB)
Dislike their appearance	7 (2.7%)	0 (0.0%)	60 (8.6%)	5 (6.0%)	13.99	**0.007**	“Personally, I think that the dogs look strange. For example Puggles” – Participant 520 (PB) “I don’t think any mix breed with a short muzzle looks nice. I’ve seen a puggle and a griffon mix breed. Just no sorry so ugly” – Participant 1209 (PNB)
Prefer a specific breed	43 (16.3%)	2 (4.9%)	57 (8.1%)	17 (7.3%)	13.672	**0.003**	“I love the Cavalier King Charles Spaniel and will only ever own that breed” – Participant 924 (PB) “I am familiar with the breed I have owned for 40 years, and see no reason to change.” – Participant 3298 (PNB)
Prefer other types of dog	0 (0.0%)	0 (0.0%)	107 (15.3%)	31 (13.2%)	51.287	**<0.001**	“Don’t appeal to me” – Participant 178 (PNB) “Not the sort of dog I would be looking for” – Participant 215 (XNB)
Don’t intend to own more dogs	4 (1.5%)	6 (14.6%)	2 (0.3%)	1 (0.4%)	78.183	**<0.001**	“Due to my age I won’t be getting another dog.” – Participant 234 (PNB)

## Discussion

The current study found crossbreeding, and specifically between a brachycephalic breed with a non-brachycephalic breed, to evoke a range of both positive and negative perceptions both within individuals as well as at a population level. These included both barriers and facilitators towards promoting this historically common, but more recently discouraged, breeding strategy as a mechanism to increase genetic diversity, reduce breed-related health disorders and improve the welfare of dogs in the future.

### Impact on health

Non-specific improvements to health were commonly described as a perceived positive consequence of crossbreeding in general. A range of specific brachycephaly-related health improvements were also perceived by many participants as potential benefits of crossbreeding between brachycephalic and non-brachycephalic dogs. These included perceptions of reduced problems with breathing, facilitated by a relatively lengthened muzzle, albeit owners of purebred brachycephalic dogs were significantly less likely to state a longer muzzle as a positive outcome of crossbreeding. Although these positive health perceptions are founded on minimal direct comparative evidence [44], the relationship between extreme conformation and risk of BOAS is well understood [19,59] and may act as facilitators of future crossbreeding initiatives.

However, perceptions were not universally positive, with the addition of the brachycephalic phenotype into a non-brachycephalic progenitor breed perceived by some participants to have potential deleterious health consequences compared to the typical health of the non-brachycephalic breed. There is currently limited evidence as to the health of brachycephalic crossbreeds compared to their purebred progenitor, although some potential has been demonstrated for improved respiratory function [44], and none compared to their non-brachycephalic progenitor. The dichotomy as to whether crossbreeding a brachycephalic breed with a non-brachycephalic breed is a force for good (by potentially improving health of the offspring compared to the brachycephalic parent) or for bad (by potentially deteriorating the health of the offspring compared to the non-brachycephalic parent) is of high importance in ongoing discussions and policy making focused on extreme conformation. Although many relevant bodies strongly recommend against acquiring a brachycephalic breed, such as the Brachycephalic Working Group’s slogan “Stop and think before buying a flat-faced dog” [60] and Dier & Recht’s (Netherlands) campaign “Don’t buy a flat-faced dog” [61], policy and advice surrounding brachycephalic crossbreeds is less clear, but developing. In the Netherlands, Dier & Recht’s ‘Healthy nose, Happy dog’ campaign featured images of extreme brachycephalic breeds such as the Pug and French Bulldog face-to-face with a markedly more moderate version of their breed, assumed to be a crossbreed given their phenotype would be unachievable at a population level within the typically extreme range of the purebreds of these breeds [62]. Understanding public perceptions of these more moderate crosses, as well as generating a greater evidence-base regarding their relative health and welfare status is of importance to facilitate evidence-based recommendations and policy.

When the scope of questioning was broadened, and participants considered the crossbreeding of any purebred dog breed with any other purebred dog breed, improvements to offspring general health were commonly cited, alongside the potential to reduce breed-specific disease risk. With growing evidence that aspects of pedigree dog breeding are negatively impacting canine health [13–17], the current study shows that dog owners are aware of and concerned about disorders linked with specific breeds. It has previously been demonstrated that owners’ perceptions of designer crossbreed dogs as healthier than purebred dogs has been a driver for increases in their popularity [47]. Epidemiological studies have shown that longevity varies widely between breeds [63]; however, longevity of crossbreed dogs exceeds that of purebred dogs [64]. Recent research has shown that the health profile of ‘designer’ Poodle crosses does not differ markedly from their purebred progenitor [43], and thus the scale of the positive health perceptions considered by participants may be unfounded. However, this research was based upon a progenitor breed which does not display extreme conformation, and it has been shown that longevity is significantly negatively affected by extreme conformation [64]. Participants in the current study showed divided opinions on whether crossbreeding (regardless of the progenitor breeds) has a positive or negative effect on dog health. This may reflect the lack of wider conclusive evidence on how the health of crossbreed dogs differs to purebred dogs (which is likely strongly influenced by marked differences in the health of individual purebreds) and other underlying concerns of dog owners regarding crossbreeding.

### Genetic diversity and disorder screening

Alongside potential health benefits gained from moving away from extreme conformation, health benefits from increased genetic diversity were recognised by participants from all ownership groups as a potential positive of crossbreeding. However, using crossbreeding as a means towards diversifying purebred gene pools was not universally accepted, with the reduction of genetic purity and potential loss of favoured breed traits, or even the extinction of certain breeds, stated as potential negatives of crossbreeding – particularly by owners of brachycephalic dogs. The concept of absolute breed purity as a desirable goal in dog breeding is thought to have arisen around the nineteenth century, linked to the ideal of a ‘superior strain’ similarly attributed to human aristocracy and thoroughbred horses. By breeding the current concept of what was the best within each breed to the best, the purebred stock was believed to be kept undiluted from external, lesser genetic influences [65]. However, in a paradigm shift supported by the growth of population genetics as a science, the practice of controlling inbreeding within restricted populations is now recognised to be harmful, rather than positive, as inbreeding increases the frequency of homozygosity, therefore increasing the chance of breeding dogs with inherited defects [66,67]. Maintaining genetic diversity within breeds is now accepted as essential to restrict the expression of deleterious genes to low levels through natural selection pressures [68].

Another barrier to widespread acceptance of crossbreeding revealed in the current study included a perception that a lack of dedicated formal ‘health testing’ (i.e., disorder testing) and health/disorder monitoring schemes for crossbred dogs would harm the health of these dogs. For example, UK Royal Kennel Club schemes such as the Respiratory Functional Grading Scheme [69] which is currently limited to three purebred brachycephalic breeds (Pug, French Bulldog and English Bulldog) though further breeds with extreme brachycephalic conformation may be added in the future. Formal recognition of newly invented designer crossbreed breeds and registration of these crossbreed dogs by national and international dog registration bodies such as the UK Royal Kennel Club and Federation Cynologique Internationale (FCI) and validation of health screening tools to include crossbreeds would allow these new types of dogs to enter existing disorder monitoring schemes. This would support the collection of more health data for these new breeds, and facilitate more controlled and monitored crossbreeding, allaying some of the current fears identified. Formal recognition and registration of crossbreeds with registration bodies such as The UK Royal Kennel Club could also increase acquisition from those people to whom pedigree and/or purebred status is desirable. Free-text analysis in the current study revealed purebred loyalty as a barrier to crossbreed acquisition. Owners of both brachycephalic and non-brachycephalic purebred dogs were more likely than owners of crossbreed dogs to state that they would only consider acquiring purebred dogs, with the status of owning a purebred well-documented as a strong motivator in acquisition [1–3]. Legitimising crossbreeding as an acceptable and laudable practice within breeding lines to maintain phenotypic and genetic diversity, as seen internationally in programs in Norway [70] and Finland [71], could help to reshape public opinion that is largely imprisoned within a purebred mantra widely promulgated as breeding excellence over the past half century.

### Predictability

Reduced predictability of looks and temperament in the offspring from crossing between a brachycephalic breed crossed with a non-brachycephalic breed was stated as a negative by around 1 in 10 participants of all ownership types. Perceptions of unpredictability in the offspring were also common amongst the reasons why participants would not consider acquiring a crossbreed brachycephalic dog. Predictability in health and temperament has been reported as a highly desirable trait for dog owners [1], while understanding and meeting owners’ expectations of their dogs is reported as an important factor in reducing relinquishment [72]. Given the high health burden in current extreme brachycephalic dogs, predictability for these breeds likely reflects that their risk of conformation-related disorders is high, although normalisation of the signs of poor health may lead to underestimation of this disease burden amongst owners [73–75]. Evidence regarding the relative health status of their crossbreed offspring is urgently needed to help the public understand whether there truly is a predictable lowering of disease burden in certain crossbreed dogs compared to purebred dogs.

### Behaviour/temperament

There was wide disagreement across participants in the current study on the potential effects of crossbreeding on the temperament of the next generation of dogs, again showing how the lack of good evidence on these issues is hampering welfare progress for dogs. Owners of crossbreed brachycephalic dogs were most likely to perceive temperament as improving as a result of crossbreeding between a brachycephalic breed with a non-brachycephalic breed. Furthermore, current owners of non-brachycephalic designer crossbreeds were most likely to perceive an improved temperament as a positive result of crossing any purebred with another purebred. These beliefs could reflect their positive experiences of the behaviour of their own dogs that were therefore influencing their perceptions of wider crossbreeding as a positive mechanism to improve temperament. Beliefs around temperament have been demonstrated as an important factor in acquisition [76–78] and previous research has reported a key reason why current owners recommend their brachycephalic breeds to others was their “comical, clown-like” personalities [52] as well as their placid temperament, low exercise needs (i.e., exercise ability) and perceived safety around children [52]. Crossbred brachycephalic dogs could therefore be better received by potential owners who value the characteristics of current extreme brachycephalic breeds if some of the perceived personality and temperament traits of extreme brachycephalic breeds could be retained in the crossbreeds, with the exception of ‘laziness’ if this reflects poor health. Consequently, to meet these human demands, priority could be given to temperament during any crossbreeding decisions such as which breeds to cross with and which dogs within individual crossbreeding programmes are selected for breeding. Better evidence on the temperament of brachycephalic crosses relative to purebred brachycephalic progenitors, and indeed potential non-brachycephalic progenitors, is needed to provide an evidence-base for both crossbreeding decisions and recommendations to prospective owners.

### Shedding and hypoallergenicity

Owners of crossbreed and non-brachycephalic dogs both perceived an advantage of crossbreeding as a lack of hair shedding and/or having less effect on owner allergies. This concept was more common in answers to the question about breeding any two purebred dogs together, but also appeared as a positive for crossbreeding a brachycephalic dog with a non-brachycephalic dog. This concept of ‘hypoallergenic’ dogs has been identified in previous studies, with a UK survey showing almost half of people acquiring a designer crossbreed with a Poodle progenitor stated a desire for a hypoallergenic dog [47]. However, there is little evidence supporting hypoallergenicity in dogs, with no difference in canine allergen levels detected between homes with reported ‘hypoallergic’ breeds or crossbreeds compared to other breeds [79]. Potential misconceptions around hair shedding and effect on human allergies is of concern, as risk of relinquishment rises when owner expectations are not met [72]. As much of the current evidence is based around Poodle-crosses, improving the evidence-base on physical characteristics of brachycephalic crosses would help provide accurate information to people interested in acquisition.

### Appearance

Physical appearance is well documented as an important factor in dog acquisition decision-making generally [77,80,81], and even more so for owners of brachycephalic breeds, where appearance has been documented as a stronger motivator for acquisition than health or longevity [51]. Some of this motivation is likely driven by the biological effects of the baby schema facial appearance that has recently been documented to apply to brachycephalic dogs [82,83] alongside potential cultural drivers such as their prominence in the media and fashion [29,84,85]. Perceived detrimental effects on the desirability of their looks following crossbreeding a brachycephalic breed with a non-brachycephalic breed was of significantly greater concern to owners of purebred brachycephalic dogs compared to owners of crossbreed or non-brachycephalic dogs. Furthermore, appearance was not a prominent barrier to the acceptance of crossbreeding more generally involving dogs of any purebred. This implies that appearance is more critical in acquisition decisions around brachycephalic dogs than non-brachycephalic, and that the appearance of offspring from brachycephalic dogs crossed with non-brachycephalic dogs is likely to be of importance to their uptake. Recent research indicated that when given the free-choice of currently extreme, less extreme or super extreme versions of brachycephalic dogs generated by AI-imagery (given the relative paucity of less extreme dogs in the current population), even current owners of brachycephalic dogs showed a clear preference for the less extreme conformation over the typical and super extreme types of dog [55]. This may offer future opportunities to use crossbreeding of extreme brachycephalics with non-brachycephalic dogs to achieve these less extreme conformations, while meeting human aesthetic preferences and improving welfare.

### Ethics of crossbreeding

The concept of ‘ethical breeding’ is complex and varying, but has previously been defined as “the use of healthy animals true to their species in behaviour and physical appearance” [86] or a system which “prioritises animal health and welfare over economic benefit or mere aesthetic appearance” [87]. Analysis of the free-text responses identified recurring concepts of irresponsible and/or uneducated breeders as concerns regarding crossbreeding, including dogs being primarily bred for fashion and/or financial gain. These concerns were common to both crossbreeding involving brachycephalic dogs and for between purebreds in general, and it is possible that the latter has influenced perceptions of the former, given the recent explosion in popularity of ‘designer crossbreeds’ [4]. Evidence suggests that designer crossbreeds are more commonly acquired in non-recommended ways that facilitate the illegal puppy trade compared to purebreds, including new owners being less likely to see their puppy in person prior to acquisition, or to see their puppy with their mother or littermates [47]. The current results further suggest that some owners were concerned that crossbreeding was motivated by breeders seeking to ‘cash in’ on the designer trend, a view which is supported by evidence that designer crossbreeds have a significantly higher purchase price than purebred puppies, with over 1 in 4 being sold for>£2000 compared to 1 in 6 purebred puppies [47]. Changing perceptions of responsible crossbreeding practices would therefore be needed to improve the image of crossbred puppies, where health and welfare has been prioritised over finances or appearance. Ensuring that crossbreeding programmes with such motivations are carried out in an evidence-based, judicious manner (e.g., appropriate selection of breeds to cross, verification of health status of dams and sires), and communicating this to the public could allay some of the current concerns identified here.

Traceability of dogs could be improved through the use of legislation, enabling prospective owners to source dogs from legal breeders more readily. In December 2023, the European Commission adopted a proposal aiming to establish uniform EU rules for the welfare of dogs and cats that are bred or kept in breeding establishments, in pet shops and in shelters [88]. This legislation could ensure that minimum standards are set for the housing, care and handling of dogs bred in the EU. Strict traceability requirements, alongside automated checks when supplying, if well enforced, should make it easier for authorities to control dog breeding, and for prospective owners to verify the origin of their future pet, increasing the chances that it will be physically and emotionally healthy, given the known risks of being bred in a low-welfare and/or illegal environments [89–91].

### Drivers of acquisition

The current study identified a common set of acquisition decision-making factors across all ownership groups. The desire to own a dog who the owner perceives to enjoy being loved and to enjoy physical affection such as strokes and cuddles featured as two out of the three highest-scoring factors consistently for owners of purebred brachycephalic dogs, crossbreed brachycephalic dogs and non-brachycephalic dogs. This is similar to a recent survey of Hungarian dog owners, which found the most appreciated factors of dog ownership were petting and physical contact with their dog, and the concept of ‘unconditional love’ [92]. The perception of a dog as a good companion has previously been reported as influential in acquisition of both brachycephalic [52] and designer crossbreed [47] dogs.

In the current study, good health ranked in the top three acquisition factor for both owners of non-brachycephalic dogs and owners of crossbreed brachycephalic dogs, but not for owners of purebred brachycephalic dogs. This finding is in line with previous studies demonstrating that health is of importance to owners of designer crossbreed dogs [47] and more influential in acquisition of non-brachycephalic compared to brachycephalic dogs [52]. The importance placed on health by all ownership groups in the current study was relatively low compared to other factors of decision-making in acquisition, although statistically significant between ownership groups, which may partly explain why, despite multiple high-profile educational campaigns regarding the poor health of brachycephalic breeds [93–96], ownership levels remain high [4,97]. Health as a relatively low priority in brachycephalic dog acquisition choice was further demonstrated in the current study where more owners of purebred brachycephalic dogs considered a breed with higher care needs as preferential, compared to owners of crossbreed and non-brachycephalic dogs. This is consistent with previous research demonstrating that poor health may actually be seen as a motivator rather than a barrier to acquisition for certain owners [98]. This desire to nurture has been recognised as a key feature in ownership of brachycephalic dogs, with the brachycephalic phenotype mimicking human infantile features such as large eyes and bulging cheeks, eliciting human feelings and behaviours associated with nurture and care [52-3,81,83,99-100]. The current study strengthens this understanding, with human care factors such as a desire for a dog owners perceive to want love and affection revealed as highly influential in acquisition. Future human behaviour-change interventions aimed at reducing ownership of brachycephalic dogs need to reflect that dogs displaying low health and requiring care and nurture may be positive motivators for some owners. Kenny *et al.* [101] assessed attitudes towards brachycephalic dogs before and after an educational intervention which aimed to increase awareness of the negative health effects of brachycephaly. Those authors found that although 82.4% of owners of brachycephalic dogs taking part reported that their awareness of brachycephalic health issues was improved by the information, only 29.3% said their perception of brachycephalic dogs had been altered [101]. The current study further demonstrates that health is of motivational low priority to some dog owners, particularly to owners of purebred brachycephalic dogs.

Owners of purebred brachycephalic dogs recorded higher scores in the current study for wanting to own a popular breed than owners of crossbreed brachycephalic or purebred non-brachycephalic dogs. The concept of breed popularity and social status adds weight to theories that certain breeds of dog can be desirable as a form of fashion [102]. As awareness of breed popularity has been shown by the current study to be important to owners of brachycephalic dogs, and current ownerships trends show a rise in designer crossbreed dogs [33], it would seem logical that demand may switch from purebred dogs to ‘designer’ crossbreeds, which also show high social popularity [47].

### Future acquisitions

Around half of owners of purebred brachycephalic dogs (55.2%) indicated they would consider a brachycephalic crossbreed for future acquisition. In a similar question, 48.8% of owners of purebred brachycephalic dogs would consider acquiring another purebred brachycephalic breed. However, care should be taken when interpreting participants’ stated acquisition intentions, as what participants say in a survey does not necessarily reflect their ultimate actions (i.e., the ‘intention-behaviour gap’) [103]. As owners of purebred brachycephalic dogs are likely to desire repeat acquisition of a brachycephalic breed [53], current owners of brachycephalic dogs are an important demographic to focus on when exploring the popularity of these breeds. Owners of extreme brachycephalic breeds have recently been demonstrated to have high levels of self-reported intractability, with almost 1 in 7 stating that ‘nothing’ could dissuade them from acquiring a brachycephalic dog [75].

The levels of re-acquisition desire for the same breed were lower in the current study than in previous estimates, such as the 93.0% of owners of Pugs, French Bulldogs and English Bulldogs surveyed in 2017 [53], although the current study shows that a similar proportion desire another brachycephalic dog – either purebred or crossbred. This may reflect changes at a wider level, with some brachycephalic breeds in the UK apparently plateauing or falling in UK Royal Kennel Club registrations in recent years. French Bulldogs, which rose in proportional registrations in the Utility group from 24.1% in 2014 to 57.4% in 2021, have maintained around this level with 52.2% of Utility registrations in 2023 [97]. Pugs peaked in percentage of registrations within the Toy group in 2017 at 40.8% but have since fallen, with 20.7% in 2022 and 14.4% in 2023 [97]. In contrast, there has been a sharp rise in ownership of designer crossbreeds in recent years [4].

Results from the current study show that the demand for purebred brachycephalic dogs may be shifting. Most participants who do not currently have a dog but are considering acquiring one stated they would not opt for a purebred brachycephalic breed, with three-quarters (73.7%) choosing to strongly or moderately disagree when asked if they would acquire a purebred brachycephalic breed. In comparison, only half (51.0%) of this group indicated they strongly or moderately disagreed with considering a crossbreed brachycephalic. Thus, a potential market for crossbreed brachycephalic dogs than purebred brachycephalic dogs exists within this demographic – although caution should be taken in interpretation of these figures, as the intention-behaviour gap principle still applies [103]. Owners of brachycephalic breeds are reported to be younger and more likely to be first-time owners [52], which may reflect the possibility of increased social media influence upon this demographic [104]. Social media has recently been demonstrated to be strongly associated with brachycephalic ownership [75]. Interventional strategies targeting such prospective owners should be encouraged, as well as continued efforts to reduce the use of dogs with extreme conformation in advertising and the media to avoid exposure to problematic extreme breeds [105–107].

### Limitations

The study had some recognised limitations. Participants were split into dog ownership categories based on whether they had ever owned a brachycephalic (purebred or crossbreed) dog, with no differentiation between those who had owned one previously and now could own a non-brachycephalic dog and those with a strong loyalty to, and current ownership of, a brachycephalic breed. This may have affected the accuracy of categorisation of opinions. There was also no differentiation made between purebred and pedigree dogs in the questionnaire, which may have led to a lack of nuance in some responses.

Although ordinal in nature, Likert data in this study were analysed as continuous data, with parametric methods for analysis of this data type have deemed suitable in some circumstances, [57]. The large sample size in the current study reduced the risk of type 1 errors, and assumptions for one-way ANOVA were met: observations were random samples from the study population and were independent of each other, data were ordinal or continuous and data were plausibly normally distributed and had equal variance [56]. This study carried out multiple statistical tests and therefore there was a higher risk of some false positive results; however, as an exploratory analysis of a little-studied area of growing importance, results can generate hypotheses for confirmation in future studies. Therefore, the specific results on each individual test should be treated with caution and safer inference gained from review of several test results considered as wider groups.

The free-text questions were designed to be easily understood and therefore accessible to a range of participants, but this simplicity may have resulted in some loss of detail. Questions on the positives and negatives of crossbreeding did not specify the purpose of the stated cross – i.e., whether as an attempt to improve current breeds (outcrossing) or to make a whole new breed (designer crossbreeding). Although the examples given did use the portmanteau names associated with designer breeds, some participants may have been referring to crossbreeding generally, and some more specifically to the concept of designer crossbreeds.

This survey had a higher proportion of female participants, as is common in surveys generally [108,109], which may have influenced the perceptions captured here compared to those of the wider population they are derived from. In order to maximise dissemination and to include as varied audience as possible, the survey was distributed across a range of social media platforms and circulated by several different organisations. The proportion of survey participants who worked across the animal care sector is higher than in the general UK population, which may have skewed findings regarding the understanding and perceptions of the health concerns considered in dog breeding, with previous studies identifying differences in attitudes and behaviours relevant to canine welfare in this professional group [110,111].

## Conclusion

Public perceptions of crossbreeding are mixed with regards to whether changes in health, appearance, temperament and diversity in resulting offspring are of positive or negative value.

The divided opinions on whether crossbreeding a brachycephalic breed with a non-brachycephalic breed would have positive effects on health (e.g., by potentially improving health of the offspring compared to the brachycephalic parent) or negative (e.g., by potentially deteriorating the health of the offspring compared to the non-brachycephalic parent) reveals an urgent need for more evidence as to the health and longevity of crossbreed dogs, and particularly in crossbreed offspring with a brachycephalic parent. However, with appearance and companionship as greater concerns than health for acquirers of brachycephalic breeds, further studies should also focus on the aesthetic appeal and behavioural characteristics of brachycephalic crossbreeds compared to their purebred brachycephalic progenitor, to explore how closely they meet the preferences of current purebred brachycephalic breed owners. Formal registration of crossbreed dogs by recognised bodies could increase public acceptance of more genetically diverse dogs, and allow for the development of controlled crossbreeding schemes and disorder testing programmes. Further evidence is required on best practice in selecting suitable non-brachycephalic breeds to facilitate crosses that maximise dog welfare and result in dogs with characteristics that are appealing to current owners of extreme brachycephalic purebreds.

## Supporting information

S1 File Study Advertisement Poster. (DOCX)

S2 File Full Survey Text. (DOCX)
