# Supplementary figures and images for: “You could get the best of both breeds or the worst of both”: UK public attitudes towards crossbreeding in dogs - with a specific focus on brachycephalic dogs
Source: PLoS One. 2026 Jan 14;21(1):e0336661. doi: 10.1371/journal.pone.0336661 (PMC12803474; doi:10.1371/journal.pone.0336661)

#### **Supporting Information 1: Study Advertisement Poster**


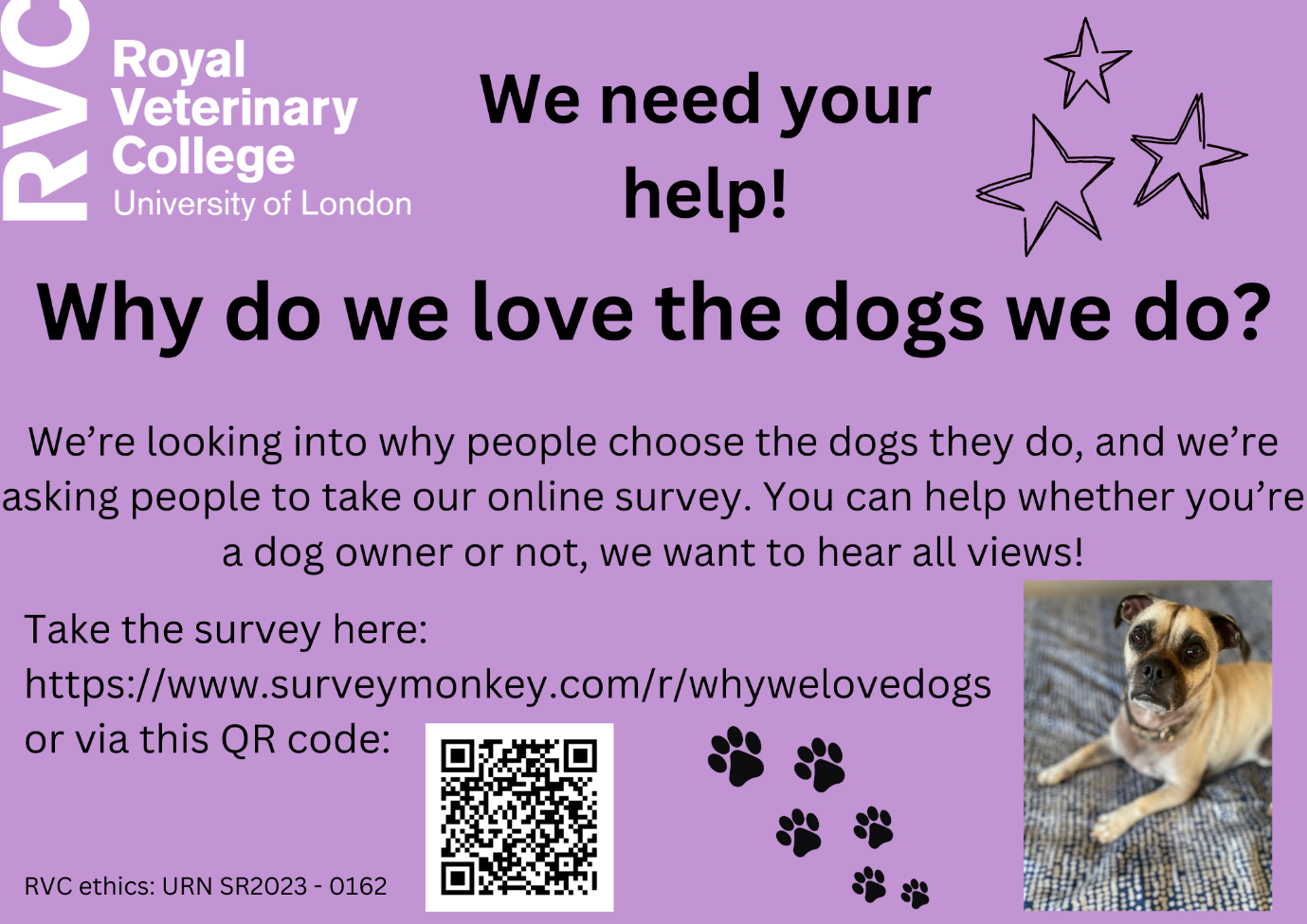

Supplement: S1 File — (DOCX) [file pone.0336661.s001.docx]
