# Supplementary material for: “You could get the best of both breeds or the worst of both”: UK public attitudes towards crossbreeding in dogs - with a specific focus on brachycephalic dogs
Source: PLoS One. 2026 Jan 14;21(1):e0336661. doi: 10.1371/journal.pone.0336661 (PMC12803474; doi:10.1371/journal.pone.0336661)
Supplement: S2 File — (DOCX) [file pone.0336661.s002.docx]

#### **Supporting Information 2: Full Survey Text**

Welcome to our survey!

**What is the survey about?**

There are now over 800 individual breeds and cross-breeds of dog in the UK for owners to choose from, each with their own unique benefits and challenges. This survey will explore your preferences for how different dog breeds and cross-breeds look, and your thoughts about various aspects of their lives, including their health and behaviour.

This survey is mostly focused on attitudes towards brachycephalic dogs (those with a shortened muzzle and/or flatter face) and their cross-breeds, but will also explore attitudes towards cross-breeding in general. You don’t need to own one of these dogs to take part. Your information will contribute to wider ongoing work to improve the welfare of dogs in the UK and is very much appreciated.

**Who can take part in this survey?**

Anyone over the age of 18 and currently resident in the UK can take part.

We welcome anyone who has any interest or views on dogs:

- Past and current dog owners
- People considering becoming dog owners in the future
- Those who have never owned a dog, and don’t intend to in the future.

**What do I need to do?**

This is an online survey, and will just take about 10-25 minutes, depending on whether you own a dog or not. We hope it will be both enjoyable, and also stimulate your thinking on dogs.

- Firstly, we would like to get a few basic details about you, including your dog ownership history. We are not asking for any identifying details, so all your responses are completely anonymised.
- Next, we will explore your thoughts about what you prefer or don’t prefer in different types of dogs, including pure-bred and cross-bred dogs.
- Finally, we will show you some pictures of dogs and ask you to rate them based on various characteristics.

**Who is conducting the research?**

The research team includes [Dr Lizzie Youens](https://www.rvc.ac.uk/about/our-people/lizzie-youens), [Dr Rowena Packer](https://www.rvc.ac.uk/about/our-people/rowena-packer) and [Dr Dan O’Neill](https://www.rvc.ac.uk/about/our-people/dan-o-neill), at the Royal Veterinary College in London, and their external collaborator, Dr Zoe Belshaw. Our research focuses on learning more about owner preferences in order to improve dog health and welfare.

**What will happen to my information?**

Your information will be stored securely by the Royal Veterinary College in accordance with general data protection regulations (GDPR). Only the research team undertaking this study will have access to your answers. We may include some of your written responses in publications or at relevant conferences but since we are not collecting any data that can identify you, nobody else would be able to recognise what you wrote.

Fully anonymised data, which cannot be traced to any individual, may be made freely available for future research and learning via the RVC Research data storage facility, and in association with subsequent publications.

**Can I change my mind and withdraw my answers?**

You are free to leave the survey at any point, up to the point of final submission. As your responses are fully anonymised, we will not be able to identify your responses from all the others later, so we are unable to identify and withdraw individual responses from the overall dataset once your responses have been submitted.

**Who is funding this research?**

This study is funded by the Animal Welfare Foundation, the RSPCA and the Blue Cross.

**What if taking part raises concerns?**

This study has received ethical approval from the Social Science Research Ethical Review Board at the Royal Veterinary College (ethics approval reference: XXXXX). If you have any questions about the study, please contact Dr. Lizzie Youens ([eyouens@rvc.ac.uk](mailto:eyouens@rvc.ac.uk)).

**I wish to take part in this research and confirm that:**

- I am 18 years old or over.
- I am currently a resident of the UK.
- I have read and understood the above information and give consent for my answers to be used for this research study and in any resulting publications.
- I give permission for the anonymised data that cannot be traced to any individual to be deposited in the RVC research data storage facility and made available in association with subsequent publications so it can be used for future research and learning.

## **Section 1: Information about you**

**1. Which age group do you fit into (years)?**

18-24

25-34

35-44

45-54

55-64

65-74

75+

Prefer not to say

**2. Which of the following best describes you?**

Male

Female

Prefer to self-describe – text box

Prefer not to say

**3. What best describes your current living situation?**

1. Live on my own

2. Live in a home with at one or more other adults (over 18 years old) and no children

3. Live in a home with one or more other adults and one or more children

4. Live in a home with one or more children where I am the only adult

5. Other (please specify) [free text]

Prefer not to say

**4. How would you describe your accommodation?**

Flat/apartment/maisonette

1-2 bedroom house/bungalow

3+ bedroom house/bungalow

Other (please specify) free text

Prefer not to say

**5. Are you or any member of your household employed in the canine and/or animal care sector (e.g. veterinary nurse, veterinary surgeon, dog groomer, dog trainer, dog behaviourist etc.)?**

1. Yes

2. No – go to Q7

3. I’m not sure

**6. Which canine and/or animal care role are you or a member of your household employed in? Please select all options that apply**

1. Veterinary surgeon
2. Veterinary nurse
3. Other veterinary staff
4. Dog behaviourist
5. Dog breeder
6. Dog trainer
7. Dog daycare/boarding kennels
8. Dog walker
9. Dog groomer
10. Rehoming centre staff
11. Other (please specify) [free text]

### **Section 2: Dog ownership history**

**7. Do you currently own or co-own one or more dogs?**

Yes– go to SECTION 3

No, but I used to own a dog– GO TO SECTION 3

No, but I am considering getting a dog in the future – GO TO SECTION 7

No, and I do not intend to get a dog in the future – go to SECTION 8

### **Section 3: Current and previous dog owners**

*In this section, we will ask some questions about your dog ownership history, and about the number and type of dogs you currently have in your household.*

**8. How many dogs do you currently own or live with?**

Drop down numbers

**9. Are you/were you the primary carer for your dog(s) (i.e. the person in your household who provides your dog with most of the care such as walking and feeding)?**

Yes

No

I share the role with someone else in the household

I share the role with someone else in a different household

**10. Did you grow up with a dog in your household?**

Yes

No

**11. As an adult, have you ever owned, or co-owned, a dog before you purchased your current/last dog?**

Yes

No, but someone in my household has

No, I am/everyone in my household is a first-time dog owner

**12. Do you currently, or have you previously, owned a *pure-bred* dog (i.e both parents are of this same breed) which is brachycephalic?**

*Brachycephalic dogs have shorter muzzles and flatter faces. Here is a list of breeds:* Affenpinscher, American Bulldog, Boston Terrier, Boxer, Bullmastiff, Chow Chow, English Bulldog, Cavalier King Charles Spaniel, Chihuahua, Dogue de Bordeaux, French Bulldog, Griffon Bruxellois, Neopolitan/Italian Mastiff, Japanese Chin, Lhasa Apso, Mastiff, Pekingese, Pomeranian, Pug, Shar Pei, Shih Tzu

Yes go to breeds

Tick all that apply

Affenpinscher, American Bulldog, Boston Terrier, Boxer, Bullmastiff, Chow Chow, English Bulldog, Cavalier King Charles Spaniel, Chihuahua, Dogue de Bordeaux, French Bulldog, Griffon Bruxellois, Neopolitan/Italian Mastiff, Japanese Chin, Lhasa Apso, Mastiff, Pekingese, Pomeranian, Pug, Shar Pei, Shih Tzu GO TO SECTION 4

No

**13. Do you currently, or have you previously owned a cross-breed dog (i.e. a mix between two or more pure-breds) whose breeding includes one of the breeds listed below?**

**If you have more than one cross-breed brachycephalic (flat-faced) breed, please answer for the dog you most recently acquired.**

Yes

Tick list – please select all breeds that are involved in your dog’s breeding

Affenpinscher, American Bulldog, Boston Terrier, Boxer, Bullmastiff, Chow Chow, English Bulldog, Cavalier King Charles Spaniel, Chihuahua, Dogue de Bordeaux, French Bulldog, Griffen Bruxellois, Italian Mastiff, Japanese Chin, Lhasa Apso, Mastiff, Old English Bulldog, Pekingese, Pomeranian, Pug, Shar Pei, Shih Tzu GO TO SECTION 5

No GO TO SECTION 6

### **SECTION 4: OWNERS OF BRACHYCEPHALIC DOGS**

*The breed you selected is classed as a ‘brachycephalic’ (flat-faced/short-nosed) dog. In this section, we will ask some questions about your dog, and whether you have ever bred from them.*

**If you have more than one dog, please think about your brachycephalic (flat-faced) dog when answering the following questions. If you have more than one brachycephalic (flat-faced) dog, please think about the one you most recently acquired.**

**14. How did you acquire your dog?**

From a breeder

I bred the dog myself

Private rehome

Rescue organisation

Other - text

**15. How many dogs of this breed have you owned, past and present?**

1

2

3

4

5

6 more

**16. Have you ever bred from one or more of these flat-faced (brachycephalic) breeds?**

*List of breeds:* Affenpinscher, American Bulldog, Boston Terrier, Boxer, Bullmastiff, Chow Chow, English Bulldog, Cavalier King Charles Spaniel, Chihuahua, Dogue de Bordeaux, French Bulldog, Griffen Bruxellois, Italian Mastiff, Japanese Chin, Lhasa Apso, Mastiff, Old English Bulldog, Pekingese, Pomeranian, Pug, Shar Pei, Shih Tzu

Yes GO TO SECTION 4A

No, but would consider it GO TO SECTION 4B

No, and would not consider it GO TO SECTION 4B

Prefer not to say GO TO SECTION 4B

### **Section 4A: BREEDERS OF BRACHYCEPHALIC DOGS**

*In this section, we will ask about why you chose your dog, your expectations for them and how those have been met, and some questions about cross-breeding dogs and your thoughts on this.*

**If you have more than one dog, please think about your brachycephalic (flat-faced) dog when answering the following questions. If you have more than one brachycephalic (flat-faced) dog, please think about the one you most recently acquired.**

**17. Do you belong to the breed club for one or more flat-faced (brachycephalic) breeds?**

Yes

No, but a member of my household does

No

Unsure

Prefer not to say

**18. Have you, or anyone in your household, ever entered any of your dogs in a dog show? By show, we are referring to a breed show rather than a novelty show, local fete etc**

Yes, once

Yes, more than once

No, but would consider it

No, and would not consider it

Prefer not to say

**If you have more than one flat-faced (brachycephalic) dog, please think about the one you most recently acquired for the next questions**

**19. Why did you choose this breed?**

ALL QUESTIONS LIKERT SCALE STRONGLY AGREE TO STRONGLY DISAGREE

I wanted a breed I’d owned before
I wanted a dog like one I’d had while growing up

I wanted a dog like one that friends or family had

I wanted a dog with a low purchase cost
I wanted a dog that had low upkeep costs

I wanted a dog I liked the look of

I wanted a dog that other people liked the look of
I wanted a dog that didn’t need much exercise

I wanted a dog who needed lots of exercise
I wanted a dog who would be safe with children
I wanted a dog whose size was suitable for my home/garden
I wanted a dog which would be healthy

I wanted a popular breed

I wanted a dog with a longer life expectancy

I wanted a dog with a shorter life expectancy

I wanted a dog which would be easy to train

I wanted a dog which would be easy to look after

I wanted a dog with high care needs

I wanted a dog which is calm

I wanted a dog which is excitable

I wanted a dog which would make me laugh

I wanted a dog who enjoys being loved

I wanted a dog who enjoys strokes and/or cuddles

I wanted a dog who would reflect who I am to others

I wanted a pedigree dog

**20. Has your dog matched the expectations you had prior to when you acquired them?**

ALL QUESTIONS LIKERT SCALE: definitely matched expectations to did not match expectations at all

Upkeep cost

Looks

How others think it looks
Exercise needs
Safety around children
Size
Health

Breed popularity

Trainability

Ease of maintenance

Calmness

Excitability

How much they make me laugh

How much they enjoy being loved

How much they enjoy strokes and/or cuddles

How they reflect who I am to others

**28. What, if any, would you consider to be the *positives* of cross-breeding any pure-bred flat-faced (brachycephalic) dog with any other pure-bred non-flat-faced (not brachycephalic) breed? E.g. a Pug crossed with a Jack Russell Terrier to make a Jug**

Free text

**29. What, if any, would you consider to be the *negatives* of cross-breeding a pure-bred flat-faced (brachycephalic) dog with any other pure-bred non-flat-faced (not brachycephalic) breed?**

Free text

**30. What, if any, would you consider to be the *positives* of cross breeding ANY pure-bred dog (i.e. not limited to brachycephalic dogs) with a dog from another breed? E.g. a Cocker Spaniel with a Poodle to make a Cockerpoo**

Free text

**31. What, if any, would you consider to be the *negatives* of cross breeding ANY pure-bred dog (i.e. not limited to brachycephalic dogs) with a dog from another breed?**

Free text

**32. Would you consider owning a different pure-bred flat-faced (brachycephalic) dog breed in the future?**

Yes, I currently own one or more other brachycephalic breeds

Yes, I would consider it

No, I would not consider it

Unsure

**33. Would you consider owning a cross-breed dog, where one parent breed is a flat-faced (brachycephalic) breed, in the future?**

Yes, I currently own a cross-breed brachycephalic dog

Yes, I would consider it

No, I would not consider it

Unsure

**Please use this space to explain your answer above, in your own words**

Free Text

**34. Are you happy to look at some pictures of dogs and answer a few questions about your opinions of them?**

Yes GO TO SECTION 8

No GO TO EXIT

### **Section 4b: non-breeders**

*In this section, we will ask about why you chose your dog, your expectations for them and how those have been met, and some questions about cross-breeding dogs and your thoughts on this.*

**If you have more than one brachycephalic (flat-faced) dog, please think about the one you most recently acquired for the next questions**.

**30. Why did you choose this breed?**

ALL QUESTIONS LIKERT SCALE STRONGLY AGREE TO STRONGLY DISAGREE

I wanted a breed I’d owned before
I wanted a dog like one I’d had while growing up

I wanted a dog like one that friends or family had

I wanted a dog with a low purchase cost
I wanted a dog that had low upkeep costs

I wanted a dog I liked the look of

I wanted a dog that other people liked the look of
I wanted a dog that didn’t need much exercise

I wanted a dog who needed lots of exercise
I wanted a dog who would be safe with children
I wanted a dog whose size was suitable for my home/garden
I wanted a dog which would be healthy

I wanted a popular breed

I wanted a dog with a longer life expectancy

I wanted a dog with a shorter life expectancy

I wanted a dog which would be easy to train

I wanted a dog which would be easy to look after

I wanted a dog which is calm

I wanted a dog which is excitable

I wanted a dog which would make me laugh

I wanted a dog who enjoys being loved

I wanted a dog who enjoys strokes and/or cuddles

I wanted a dog who would reflect who I am to others

I wanted a pedigree dog

**31. Has your dog matched the expectations you had prior to when you acquired them?**

ALL QUESTIONS LIKERT SCALE: definitely matched expectations to did not match expectations at all

Purchase cost
Upkeep cost

Looks

How others think it looks
Exercise needs
Safety around children
Size
Health

Breed popularity

Trainability

Ease of maintenance

Calmness

Excitability

How much they make me laugh

How much they enjoy being loved

How much they enjoy strokes and/or cuddles

How they reflect who I am to others

**21. What, if any, would you consider to be the *positives* of cross-breeding any pure-bred flat-faced (brachycephalic) dog with any other pure-bred non-flat-faced (not brachycephalic) breed? E.g. a Pug crossed with a Jack Russell Terrier to make a Jug**

Free text

**22. What, if any, would you consider to be the *negatives* of cross-breeding a pure-bred flat-faced (brachycephalic) dog with any other pure-bred non-flat-faced (not brachycephalic) breed?**

Free text

**23. What, if any, would you consider to be the *positives* of cross breeding ANY pure-bred dog (i.e. not limited to brachycephalic dogs) with a dog from another breed? E.g. a Cocker Spaniel with a Poodle to make a Cockerpoo**

Free text

**24. What, if any, would you consider to be the *negatives* of cross breeding ANY pure-bred dog (i.e. not limited to brachycephalic dogs) with a dog from another breed?**

Free text

**38. Do you think your breed of dog should be crossed with another breed?**

**LIKERT SCALE: STRONGLY AGREE TO STRONGLY DISAGREE**

**39. If your breed of dog was to be crossed with another breed, what changes do you think would be acceptable?**

LIKERT SCALE: VERY HAPPY TO CHANGE TO VERY UNHAPPY TO CHANGE

Looks

Trainability

Personality

Health

Longevity

Independence

Sociability

Being a pedigree

**32. If you were to get another dog, would you own the same breed again?**

Yes

No

Unsure

**40. Would you consider owning a different pure-bred flat-faced (brachycephalic) dog breed in the future?**

Yes, I currently own one or more other brachycephalic breeds

Yes, I would consider it

No, I would not consider it

Unsure

**41. Would you consider owning a cross-breed dog where one parent breed is a flat-faced (brachycephalic) breed, in the future?**

Yes, I currently own a cross-breed brachycephalic dog

Yes, I would consider it

No, I would not consider it

Unsure

**Please use this space to explain your answer above, in your own words**

Free Text

**42. Are you happy to look at some pictures of dogs and answer a few questions about your opinions of them?**

Yes GO TO SECTION 8

No GO TO EXIT

### **SECTION 5: OWNERS OF CROSS-BREED BRACHYCEPHALIC DOGS**

*In this section, we will ask about why you chose your dog, your expectations for them and how those have been met, and some questions about cross-breeding dogs and your thoughts on this.*

**If you have more than one dog, please think about your dog which has a brachycephalic (flat-faced) parent breed when answering the following questions. If you have more than one flat-faced cross-breed dog, please think about the one you most recently acquired.**

**43. How did you acquire your dog?**

From a breeder

I bred the dog myself

Private rehome

Rescue organisation

Other - text

**44. Why did you choose this cross-breed?**

ALL QUESTIONS LIKERT SCALE STRONGLY AGREE TO STRONGLY DISAGREE

I wanted a dog like one I’d owned before
I wanted a dog like one I’d had while growing up

I wanted a dog like one that friends or family had

I wanted a dog with a low purchase cost
I wanted a dog that had low upkeep costs

I wanted a dog I liked the look of

I wanted a dog that other people liked the look of
I wanted a dog that didn’t need much exercise

I wanted a dog who needed lots of exercise
I wanted a dog who would be safe with children
I wanted a dog whose size was suitable for my home/garden
I wanted a dog which would be healthy

I wanted a popular cross-breed

I found the name of this cross-breed appealing

I wanted a dog with a longer life expectancy

I wanted a dog with a shorter life expectancy

I wanted a dog which would be easy to train

I wanted a dog which would be easy to look after

I wanted a dog which is calm

I wanted a dog which is excitable

I wanted a dog which would make me laugh

I wanted a dog who enjoys being loved

I wanted a dog who enjoys strokes and/or cuddles

I wanted a dog who would reflect who I am to others

I wanted a pedigree dog

**45. Has your dog matched the expectations you had prior to when you acquired them?**

ALL QUESTIONS LIKERT SCALE: definitely matched expectations to did not match expectations at all

Purchase cost
Upkeep cost

Looks

How others think it looks
Exercise needs
Safety around children
Size
Health

Cross-breed popularity

Trainability

Ease of maintenance

Calmness

Excitability

How much they make me laugh

How much they enjoy being loved

How much they enjoy strokes and/or cuddles

How they reflect who I am to others

**46. Did you deliberately choose a cross-breed flat-faced (brachycephalic) dog over a pure-bred brachycephalic breed?**

Yes

No – skip next question

**Please use this space to explain why, in your own words**

Free text

**Please look at the statements below, and respond with how much you agree with each statement in relation to your cross-breed brachycephalic dog.**

**47. My dog having a brachycephalic breed in its parentage was important to me**

Likert strongly disagree to strongly agree

**48. I am happy with my decision to acquire a cross-breed dog rather than a pure-breed dog**

Likert strongly agree to strongly disagree

**21. What, if any, would you consider to be the *positives* of cross-breeding any pure-bred flat-faced (brachycephalic) dog with any other pure-bred non-flat-faced (not brachycephalic) breed? E.g. a Pug crossed with a Jack Russell Terrier to make a Jug**

Free text

**22. What, if any, would you consider to be the *negatives* of cross-breeding a pure-bred flat-faced (brachycephalic) dog with any other pure-bred non-flat-faced (not brachycephalic) breed?**

Free text

**23. What, if any, would you consider to be the *positives* of cross breeding ANY pure-bred dog (i.e. not limited to brachycephalic dogs) with a dog from another breed? E.g. a Cocker Spaniel with a Poodle to make a Cockerpoo**

Free text

**24. What, if any, would you consider to be the *negatives* of cross breeding ANY pure-bred dog (i.e. not limited to brachycephalic dogs) with a dog from another breed?**

Free text

**49. If you were to get another dog, would you own the same cross-breed again?**

Yes

No

Unsure

**Please explain your answer, in your own words**

Free text

**56. Would you own a different cross-bred flat-faced (brachycephalic) dog breed in the future?**

Yes, I already own a different cross-breed brachycephalic dog

Yes, I would consider it

No, I would not consider it

Unsure

**Please use this space to explain your answer above, in your own words**

Free text

**57. Would you consider owning a pure-bred brachycephalic (flat-faced) dog in the future?**

Yes, I already own a pure-bred brachycephalic breed

Yes, I would consider it

No, I would not consider it

Unsure

**Please use this space to explain your answer above, in your own words**

Free Text

**58. Are you happy to look at some pictures of dogs and answer a few questions about your opinion of them?**

Yes GO TO SECTION 8

No GO TO EXIT

### **SECTION 6: OWNERS OF NON-BRACHYCEPHALIC DOGS**

*In this section, we will ask about why you chose your dog, your expectations for them and how those have been met, and some questions about cross-breeding dogs and your thoughts on this.*

**If you have more than one dog, please think about the one you most recently acquired.**

**59. Is your dog a pure-bred breed (i.e., where both parents are the same breed and may or may not be registered with a registration body such as the Kennel Club) or a cross-breed (i.e. a mix between two or more pure-breds)?**

Pure-bred

Cross-breed – skip next question

Unsure

**60. Which breed is your dog?**

Free text – then skip next question

**61. What is your dog’s cross?**

**(Please state the known cross if possible, e.g. ‘Cocker Spaniel x Poodle – Cockapoo’, or state any of the breeds included in your dog’s cross if unknown, e.g. ‘Labrador cross’**

Free text

**62. How did you acquire your dog?**

From a breeder

I bred the dog myself

Private rehome

Rescue organisation

Other - text

**63. Why did you choose this breed?**

ALL QUESTIONS LIKERT SCALE STRONGLY AGREE TO STRONGLY DISAGREE

I wanted a breed I’d owned before
I wanted a dog like one I’d had while growing up

I wanted a dog like one that friends or family had

I wanted a dog with a low purchase cost
I wanted a dog that had low upkeep costs

I wanted a dog I liked the look of

I wanted a dog that other people liked the look of
I wanted a dog that didn’t need much exercise

I wanted a dog who needed lots of exercise
I wanted a dog who would be safe with children
I wanted a dog whose size was suitable for my home/garden
I wanted a dog which would be healthy

I wanted a popular breed

I wanted a dog with a longer life expectancy

I wanted a dog with a shorter life expectancy

I wanted a dog which would be easy to train

I wanted a dog which would be easy to look after

I wanted a dog which is calm

I wanted a dog which is excitable

I wanted a dog which would make me laugh

I wanted a dog who enjoys being loved

I wanted a dog who enjoys strokes and/or cuddles

I wanted a dog who would reflect who I am to others

I wanted a pedigree dog

**64. Has your dog matched the expectations you had prior to when you acquired them?**

ALL QUESTIONS LIKERT SCALE: definitely matched expectations to did not match expectations at all

Purchase cost
Upkeep cost

Looks

How others think it looks
Exercise needs
Safety around children
Size
Health

Breed popularity

Trainability

Ease of maintenance

Calmness

Excitability

How much they make me laugh

How much they enjoy being loved

How much they enjoy strokes and/or cuddles

How they reflect who I am to others

**21. What, if any, would you consider to be the *positives* of cross-breeding any pure-bred flat-faced (brachycephalic) dog with any other pure-bred non-flat-faced (not brachycephalic) breed? E.g. a Pug crossed with a Jack Russell Terrier to make a Jug**

Free text

**22. What, if any, would you consider to be the *negatives* of cross-breeding a pure-bred flat-faced (brachycephalic) dog with any other pure-bred non-flat-faced (not brachycephalic) breed?**

Free text

**23. What, if any, would you consider to be the *positives* of cross breeding ANY pure-bred dog (i.e. not limited to brachycephalic dogs) with a dog from another breed? E.g. a Cocker Spaniel with a Poodle to make a Cockerpoo**

Free text

**24. What, if any, would you consider to be the *negatives* of cross breeding ANY pure-bred dog (i.e. not limited to brachycephalic dogs) with a dog from another breed?**

Free text

**65. If you were to get another dog, would you own the same breed or cross-breed again?**

Yes

No

Unsure

**72. Would you consider owning a pure-bred brachycephalic (flat-faced) dog in the future?**

Yes, I already own a pure-bred brachycephalic dog

Yes, I would consider it

No, I would not consider it

Unsure

**73. Please use this space to explain your answer above, in your own words**

Free Text

**74. Would you consider owning a cross-bred flat-faced (brachycephalic) dog breed in the future?**

Yes, I already own a cross-bred brachycephalic dog

Yes, I would consider it

No, I would not consider it

Unsure

**Please use this space to explain your answer above, in your own words**

Free text

**75. Are you happy to look at some pictures of dogs and answer a few questions about your opinions of them?**

Yes GO TO SECTION 8

No GO TO EXIT

### **Section 7: Prospective owners**

**76. Do you have a particular breed/cross-breed or type in mind for your future puppy/dog?**

Yes – I have a particular breed/cross-breed or type in mind

We want a rescue, but have some preference regarding breed or type

We want a rescue, and have no preference regarding breed or type skip next question

No, I do not have a preference skip next question

I/we are currently trying to decide

Don’t know

**77. Are you considering acquiring a pure-breed dog or a cross-breed dog?**

Pure-breed dog

Cross-breed dog skip next question

I am considering both pure-breeds and cross-breeds

Unsure

**79. How likely are you to acquire a pure-bred brachycephalic breed?**

*A brachycephalic breed is one with a shorter muzzle and/or a flatter face.*

*Here are a list of brachycephalic breeds: Affenpinscher, American Bulldog, Boston Terrier, Boxer, Bullmastiff, Chow Chow, English Bulldog, Cavalier King Charles Spaniel, Chihuahua, Dogue de Bordeaux, French Bulldog, Griffon Bruxellois, Neopolitan/Italian Mastiff, Japanese Chin, Lhasa Apso, Mastiff, Pekingese, Pomeranian, Pug, Shar Pei, Shih Tzu*

Likert scale extremely likely to extremely unlikely

**80. How likely are you to acquire a cross-bred brachycephalic breed?**

Likert scale extremely likely to extremely unlikely

**81. What traits are important when choosing a dog?**

ALL QUESTIONS LIKERT SCALE STRONGLY AGREE TO STRONGLY DISAGREE

I want a breed I’d owned before
I want a dog like one I’d had while growing up

I want a dog like one that friends or family had

I want a dog with a low purchase cost
I want a dog that had low upkeep costs

I want a dog I liked the look of

I want a dog that other people liked the look of
I want a dog that didn’t need much exercise

I want a dog who needed lots of exercise
I want a dog who would be safe with children
I want a dog whose size was suitable for my home/garden
I want a dog which would be healthy

I want a popular breed

I want a dog with a longer life expectancy

I want a dog with a shorter life expectancy

I want a dog which would be easy to train

I want a dog which would be easy to look after

I want a dog which is calm

I want a dog which is excitable

I want a dog which would make me laugh

I want a dog who enjoys being loved

I want a dog who enjoys strokes and/or cuddles

I want a dog who would reflect who I am to others

I want a pedigree dog

**82. Are you happy to look at some pictures of dogs and answer a few questions about them?**

Yes GO TO SECTION 8

No GO TO EXIT

### **Section 8: What do you think of these dogs?**

In this section, we would like you to look at three sets of pictures of four dogs, and answer some questions about them. There are five or six questions for you to answer about each set of dogs, after you have examined the pictures.

### **SET 1**

**Look carefully at the dogs shown in the following FOUR pictures, and consider these questions:**

Pictures of Pugs:

1. Very extreme pug
2. Extreme pug
3. More moderate pug

**83. Which of these dogs makes you feel happiest when you look at them?**

**Please rank from 1 (makes me most happy) to 4 (makes me least happy), or use the options below if they all have an equal effect on you**

1-4 RANK

They all make me feel equally happy

They all make me feel equally unhappy

**84. Which of these dogs looks the most attractive?**

**Please rank from 1 (most attractive) to 4 (least attractive), or use the options below if they all have an equal effect on you**

1-4 RANK

I think they all look equally attractive

I think they all look equally unattractive

**85. Which of these dogs look the healthiest?**

**Please rank from 1 (most healthy) to 4 (least healthy), or use the options below if they all have an equal effect on you**

1-4 RANK

I think they all look equally healthy

I think they all look equally unhealthy

**86. Which of these dog(s) do you think look like they have been bred ethically – i.e. in a way that prioritises health and temperament traits over looks or financial gain?**

**Please rank from 1 (most likely to have been bred ethically) to 4 (least likely to have been bred ethically), or use the options below if they all have an equal effect on you**

1-4 RANK

I think they all equally look like they’ve been bred ethically

I think they all equally look like they’ve been bred ethically

Unsure/don’t know

**87. Which of these dogs, if any, do you think are purebred Pugs**?

1-4 CHOOSE AS MANY AS YOU LIKE

I think they all look equally like purebred Pugs

I don’t think any look like purebred Pugs

**88. Which of these dogs would you most like to own?**

**Please rank from 1 (most like to own) to 4 (least like to own), or use the options below if they all have an equal effect on you**

1-4 RANK

I would like to own all of these dogs equally

I wouldn’t like to own any of these dogs

**SET 2**

**Look carefully at the dogs shown in the following FOUR pictures, and consider these questions:**

Pictures of French Bulldogs:

1. Very extreme French Bulldog
2. Extreme French Bulldog
3. More moderate French Bulldog

**89. Which of these dogs makes you feel happiest when you look at them?**

**Please rank from 1 (makes me most happy) to 4 (makes me least happy), or use the options below if they all have an equal effect on you**

1-4 RANK

They all make me feel equally happy

They all make me feel equally unhappy

**90. Which of these dogs looks the most attractive?**

**Please rank from 1 (most attractive) to 4 (least attractive), or use the options below if they all have an equal effect on you**

1-4 RANK

I think they all look equally attractive

I think they all look equally unattractive

**91. Which of these dogs look the healthiest?**

**Please rank from 1 (most healthy) to 4 (least healthy), or use the options below if they all have an equal effect on you**

1-4 RANK

I think they all look equally healthy

I think they all look equally unhealthy

**92. Which of these dog(s) do you think look like they have been bred ethically – i.e. in a way that prioritises health and temperament traits over looks or financial gain?**

**Please rank from 1 (most likely to have been bred ethically) to 4 (least likely to have been bred ethically), or use the options below if they all have an equal effect on you**

1-4 RANK

I think they all equally look like they’ve been bred ethically

I think they all equally look like they’ve been bred ethically

Unsure/don’t know

**93. Which of these dogs, if any, do you think are purebred French Bulldogs**?

1-4 CHOOSE AS MANY AS YOU LIKE

I think they all look equally like purebred French Bulldogs

I don’t think any look like purebred French Bulldogs

**94. Which of these dogs would you most like to own?**

**Please rank from 1 (most like to own) to 4 (least like to own), or use the options below if they all have an equal effect on you**

1-4 RANK

I would like to own all of these dogs equally

I wouldn’t like to own any of these dogs

### **SET 3**

**Look carefully at the dogs shown in the following FOUR pictures, and consider these questions:**

Pictures of English Bulldogs:

1. Very extreme English Bulldog
2. Extreme English Bulldog
3. More moderate English Bulldog

**95. Which of these dogs makes you feel happiest when you look at them?**

**Please rank from 1 (makes me most happy) to 4 (makes me least happy), or use the options below if they all have an equal effect on you**

1-4 RANK

They all make me feel equally happy

They all make me feel equally unhappy

**96. Which of these dogs looks the most attractive?**

**Please rank from 1 (most attractive) to 4 (least attractive), or use the options below if they all have an equal effect on you**

1-4 RANK

I think they all look equally attractive

I think they all look equally unattractive

**97. Which of these dogs look the healthiest?**

**Please rank from 1 (most healthy) to 4 (least healthy), or use the options below if they all have an equal effect on you**

1-4 RANK

I think they all look equally healthy

I think they all look equally unhealthy

**98. Which of these dog(s) do you think look like they have been bred ethically – i.e. in a way that prioritises health and temperament traits over looks or financial gain?**

**Please rank from 1 (most likely to have been bred ethically) to 4 (least likely to have been bred ethically), or use the options below if they all have an equal effect on you**

1-4 RANK

I think they all equally look like they’ve been bred ethically

I think they all equally look like they’ve been bred ethically

Unsure/don’t know

**99. Which of these dogs, if any, do you think are purebred English Bulldogs**?

1-4 CHOOSE AS MANY AS YOU LIKE

I think they all look equally like purebred English Bulldogs

I don’t think any look like purebred English Bulldogs

**100. Which of these dogs would you most like to own?**

**Please rank from 1 (most like to own) to 4 (least like to own), or use the options below if they all have an equal effect on you**

1-4 RANK

I would like to own all of these dogs equally

I wouldn’t like to own any of these dogs

### **Exit and Further Information**

Thank you so much for contributing to a better future for all dogs by your participation in this survey, we really do appreciate your time and effort. Your answers are all hugely valuable, and will help improve dog welfare in the UK.

**Concerns about your dog’s health or wellbeing?**
If completing this survey has raised any concerns about your own dog, please contact your dog’s veterinary surgery, or register with a local veterinary practice if you have not registered your dog as yet. To find a local veterinary surgeon, please visit the following website:
[https://findavet.rcvs.org.uk/home/](https://findavet.rcvs.org.uk/home/ )

**Learn more about brachycephalic dogs**

**Flat-faced (brachycephalic) dogs suffer from a range of health problems. Learn more about affected breeds and the related health conditions here:**

- **The Royal Veterinary College’s VetCompass program:** <https://www.rvc.ac.uk/vetcompass>
- **The UK Brachycephalic Working Group:** <https://www.ukbwg.org.uk>
- **The International Collaborative on Extreme Conformations in Dogs:** <https://dogwellnet.com/icecdogs/>
- **The Blue Cross:** <https://www.bluecross.org.uk/advice/dog/things-to-think-about-before-buying-a-flat-faced-brachycephalic-dog>
- **The RSPCA:** <https://www.rspca.org.uk/adviceandwelfare/pets/brachycephalic>
- **The British Veterinary Association:** <https://www.bva.co.uk/take-action/our-policies/brachycephalic-dogs/>

**Thinking about buying a dog in the future?**There are tools available to help you identify safe and responsible methods of acquiring and new puppy or dog.

The Puppy Contract is a free tool designed by the All Parliamentary Group for Animal Welfare, the Animal Welfare Foundation, the Blue Cross, the British Veterinary Association, DEFRA, the Dogs Trust, The Kennel Club, the PDSA and the RSPCA to help pet owners in their search for a healthy, happy puppy. For more information and a free downloadable version of the contract visit <https://www.puppycontract.org.uk>

The Petfished public information campaign is a government initiative from the Department for Environment, Food and Rural Affairs (DEFRA) in England that aims to alert the public to unscrupulous breeders of puppies and kittens. Petfishing is where unscrupulous sellers pretend that the puppy they’re selling you comes from a happy home, but in reality, the animal may have been bred or kept in poor conditions. More information about the campaign is available at <https://getyourpetsafely.campaign.gov.uk>

If you have any questions about this survey, please contact Dr Lizzie Youens at eyouens@rvc.ac.uk.
